# Variations in “Functional Site” Residues and Classification of Three-Finger Neurotoxins in Snake Venoms

**DOI:** 10.3390/toxins17080364

**Published:** 2025-07-24

**Authors:** R. Manjunatha Kini, Cho Yeow Koh

**Affiliations:** 1 Department of Biological Sciences, Faculty of Science, National University of Singapore, Singapore 117558, Singapore; 2 Department of Pharmacology, Yong Loo Lin School of Medicine, National University of Singapore, Singapore 117600, Singapore; 3 Department of Biochemistry, Medical College of Virginia, Virginia Commonwealth University, Richmond, VA 23298, USA; 4 Department of Medicine, Yong Loo Lin School of Medicine, National University of Singapore, Singapore 117559, Singapore

**Keywords:** nicotinic acetylcholine receptor (nAChR), ligand-gated ion channels, molecular evolution, phylogenic analysis, sequence alignment, structure–function relationships, protein–protein interaction

## Abstract

Three-finger toxins (3FTxs) are the largest group of nonenzymatic toxins found in snake venoms. Among them, neurotoxins that target nicotinic acetylcholine receptors are the most well-studied ligands. In addition to the classical neurotoxins, several other new classes have been characterized for their structure, receptor subtype, and species selectivity. Here, we systematically analyzed over 700 amino acid sequences of three-finger neurotoxins that interact with nicotinic acetylcholine receptors. Based on the amino acid residue substitutions in the functional sites and structural features of various classes of neurotoxins, we have classified them into over 150 distinct subgroups. Currently, only a small number of typical examples representing these subgroups have been studied for their structure, function, and subtype selectivity. The functional site residues responsible for their interaction with specific receptor subtypes of several toxins are yet to be identified. The molecular details of each subgroup representative toxin with its target receptor will contribute towards the understanding of subtype- and/or interface-selectivity. Thus, this review will provide new impetus in the toxin research and pave the way for the design of potent, selective ligands for nicotinic acetylcholine receptors.

## 1. Introduction

The toxins in snake venoms have evolved to play a critical role in prey capture and predator deterrence. Among them, postsynaptic neurotoxins are important players, causing immobilization, paralysis, and death of the prey. Most of these postsynaptic neurotoxins are nonenzymatic polypeptides and belong to the three-finger toxin (3FTx) family of venom proteins [1,2,3]. They target nicotinic acetylcholine receptors (nAChRs) and bind to the same site as the endogenous agonist acetylcholine (ACh) that activates these ligand-gated ion channels. In general, these neurotoxins are monomeric proteins that interact with different nAChR subtypes. They were initially classified into two subclasses: (a) short-chain (57–62 residues) α-neurotoxins with four conserved disulfide bridges in the core region, and (b) long-chain (66–75 residues) α-neurotoxins with four conserved disulfide bridges and an additional fifth disulfide bridge at the tip of loop II [1,2,3,4]. Both short- and long-chain α-neurotoxins block muscle-type nAChRs with similar affinities (K_d_ ~10^−9^–10^−11^ M) [5], but only long-chain α-neurotoxins block neuronal α7, α9, and α9α10 nAChRs with high affinity (K_d_ ~10^−8^–10^−9^ M) [6]. These toxins were identified as Type I and Type II neurotoxins, respectively [7]. A third group of neurotoxins, κ-bungarotoxin from krait venom, was identified based on the ability to block neuronal nAChRs in the chick ciliary ganglion, which was not susceptible to α-bungarotoxin [8]. Structurally, these neurotoxins are similar to Type II neurotoxins with the fifth disulfide at the tip of loop II. Unlike monomeric Type I and Type II neurotoxins, this group of neurotoxins forms non-covalent dimers. They block α3-containing nAChRs (IC_50_ < 150 nM) but fail to block α2- and/or α4-containing nAChRs (for details, see [8]). Subsequently, four additional distinct neurotoxin groups were added: (c) Type III neurotoxins [9]; (d) non-conventional toxins (formerly, weak toxins [10,11,12]) that have the fifth disulfide bond in loop I [13,14]; (e) Ω-neurotoxins [15]; and (f) Σ-neurotoxins [16].

In addition to the 3FTx scaffold, other protein families have also evolved to target nAChRs. For example, snake venom toxins with waglerin, phospholipase A_2_, C-type lectin (snaclec), azemiopsin, and baptides families also target nAChRs. Two waglerins from *Tropidolaemus* (formerly, *Trimeresurus*) *wagleri* venom have been purified and characterized [17]. They have 24 amino acid residues and a single disulfide bond. Some phospholipases A_2_ and their complexes exhibit postsynaptic effects [18,19,20,21,22]. These enzymatic proteins have 120–125 amino acid residues and seven disulfide bonds. Recently, Utkin et al. isolated a new class of antagonist from *Azemiops feae* viper venom and named it azemiopsin [23]. It has 21 amino acid residues and no cysteine residues or disulfide bonds. Interestingly, the C-terminal segments of waglerins and azemiopsin exhibit significant similarities [23]. Similar proline-rich peptides from *Psammophis mossambicus* are antagonists of α7 nAChRs [24]. Snaclecs, banarecin from *Bitis arietans* and emunarecins from *Echis mucrosquamatus* venoms, inhibit the currents induced in *Lymnaea stagnalis* neurons through a mechanism independent of lectin-like function [25]. Vulvius et al. also purified baptides from *Bitis arietans* venom that reversibly block nAChRs [26]. Baptide 1 has seven, while baptides 2 and 3 have ten amino acid residues. Interestingly, baptide 3 is acetylated at the N-terminus. This new class of antagonists is noncompetitive blockers, unlike all other antagonists described above [26]. The diversity of toxin families that have convergently evolved to target nAChRs highlights the importance of this receptor. Further details on these and other antagonists are well documented in recent reviews [1,2,6]. Here, we focus on neurotoxins that belong to the 3FTx family.

Structure–function relationships of α-neurotoxins have been well characterized, and the functional site residues that are involved in the interaction with nAChRs have been determined [27,28,29,30,31]. In this manuscript, when discussing these toxins, we have retained the original residue numbers for consistency and familiarity. However, because we performed sequence alignment using a large number of new 3FTx sequences, alignment gaps have resulted in changes to the residue numbering. To address this, we have included the new residue numbers, as established by sequence alignments, in brackets as homology numbers whenever they differ from the original. The functional sites of Type I and Type II α-neurotoxins are closely related; 10 to 12 specific functional residues, Ser^8^/—, Gln^10^/—, Lys^27^/Lys^23(27)^, Trp^29^/Trp^25(29)^, Asp^31^/Asp^27(31)^, Phe^32(36)^/Phe^29(33)^, Arg^33(37)^/Arg^33(37)^, —/Arg^36(40)^, Glu^38(42)^/—, Lys^47(53)^/Lys^49(53)^, and —/Phe^65(70)^ [erabutoxin b/α-cobratoxin (short- and long-chain neurotoxins, respectively) numbering] are critical for interaction with nAChRs [27,29]. Along with these toxin-specific residues, both long- and short-chain α-neurotoxins utilize a common binding core to interact with different nAChRs. This region consists of Lys^27^/Lys^23(27)^, Trp^29^/Trp^25(29)^, Asp^31^/Asp^27(31)^, Arg^33(37)^/Arg^33(37)^, and Lys^47(53)^/Lys^49(53)^, and establishes contact with invariant residues on the nAChRs. Mutation of the common binding core residues of α-neurotoxins causes significant (ranging from 20- to 780-fold) loss of affinity towards nAChRs [27,29]. Some of the non-conventional toxins have several of these functional residues (topologically conserved), and these toxins exhibit similar affinity towards nAChRs [13].

Interestingly, the other two classes of 3FTxs, Type III neurotoxins [9] and Ω-neurotoxins [15], lack these typical functionally important residues of canonical short- and long-chain α-neurotoxins. Using site-directed mutagenesis of Oh9-1, an Ω-neurotoxin from *Ophiophagus hannah* venom, we identified the functional site residues that allow the binding of α-neurotoxins to rat α1β1εδ and α3β2 nAChRs [15]. Interestingly, Oh9-1 uses distinct amino acid residues to bind to the two subtypes. The functional site residues in Type III neurotoxins are yet to be identified. Our understanding of structure–function relationships, subtype selectivity, and molecular recognition sites of snake venom antagonists of nAChRs is far from complete.

With the advent of new technologies, hundreds of new neurotoxins belonging to 3FTxs have been added to the database. But the details of the function of only a small number of these toxins have been documented. We focused on the distinct differences in the structures of 3FTxs and their potential impact on function. Recently, we have classified Type II neurotoxins into three classes based on the number of residues in the C-terminal tail and their conserved, functionally important residues [32]. To understand the structure–function relationships of these and other classes of 3FTxs, here we have further extended this classification. Our analyses of the cDNA sequences indicate that various classes of 3FTxs have evolved by changing functional site residues. Through these analyses, we identified several new classes of 3FTxs that may exhibit postsynaptic blockades of neurotransmission by binding to various subtypes of nAChRs. We also used structure predictions [33] to depict a few of these new classes and compared them to better known 3FTxs. We hope that this classification will provide impetus for further detailed studies on various classes to understand their specificity and selectivity towards nAChRs. Structure–function relationships of some of these novel antagonists may help us understand specific residues involved in molecular recognition and subtype selectivity.

## 2. Results and Discussion

nAChRs play an important role in neurotransmission both in the central and peripheral nervous systems [34]. They are distributed in both the postsynaptic and presynaptic sides. The postsynaptic nAChRs convert ACh released into the synaptic cleft into an ion flux, which triggers excitatory postsynaptic potentials [35,36]. However, the majority of nAChRs are presynaptic, and their activation leads to calcium influx, which in turn enhances the release of neurotransmitters [35,37,38,39]. Thus, they regulate not only ACh transmission but also transmission of glutamate, dopamine, and glycine [40,41,42,43,44].

The nAChRs are formed by pentameric assembly of closely related subunits. They can be heteromeric (adult/fetal muscle-type α1β1εδ/α1β1γδ and neuronal α3β2, α4β2, α4β4, α3β2, α3β4, and α9α10) or homomeric (neuronal α7 and α9). Although the endogenous α7 nAChRs are homopentamers, some reports indicate the existence of functional heteromeric α7β2 subtypes [45,46]. At times, they exhibit the same subunit composition but different subunit stoichiometry, giving both molecular diversity and functional heterogeneity. α7 [46,47,48] and α4β2 [35,47,49] nAChRs are major nAChR subtypes in the central nervous system. The activation of α7 upregulates the blood–brain barrier function through increased claudin-5 and occludin expression in rat brain endothelial cells [50]. After ischemic stroke, α7 reduces the damage to the blood–brain barrier by regulating inflammation and autophagy [51].

In addition to its expression in the CNS, α7 nAChRs are expressed in non-neuronal cells such as macrophages [35,52,53,54,55]. Further, compared to other neuronal subtypes, α7 nAChRs have higher calcium permeability, which in turn can induce downstream cell-signaling mechanisms [46,54,56]. Since the α7 subtype is also selectively activated by choline, a more ubiquitous precursor compared to acetylcholine [57], it is thought to be an early form of nAChR that evolved in ancestral organisms that do not rely on fast excitatory mechanisms [54]. Thus, physiological functions of α7 nAChRs extend beyond the classical excitatory neurotransmission and may mediate metabotropic signaling in non-neuronal cell types such as cells of the immune system [46,54]. The α4β2 nAChR is highly expressed in the brain [35,47,49]. It is one of the primary targets of nicotine in the brain [58] and mediates the addictive properties of nicotine [35,46]. Heterologous expression indicated two possible stoichiometric arrangements, (α4)2(β2)3 and (α4)3(β2)2, with distinct affinities; (α4)2(β2)3 exhibits high affinity, while (α4)3(β2)2 exhibits low affinity for nicotine [59,60,61]. The α3β4 and α3β2 are the major nAChR subtypes expressed in the peripheral nervous system, and they mediate autonomic functions [47,62]. The α3β4 and α3β2 subtype nAChRs can exist in different stoichiometric arrangements of α3 and β2/β4 subunits or associate with the α5 and/or α6 subunits [47]. Such variations in stoichiometry can have a profound influence on α3β4 and α3β2 pharmacological properties.

Over the last three decades, a tremendous amount of physiological, pharmacological, and structural data has accumulated from extensive research on the nAChRs. This is primarily due to the therapeutic potential associated with these receptors, placing them among the top pharmaceutical targets [63,64]. Neuronal nAChRs are involved in regulatory processes (neurotransmitter release, cell excitability, and neuronal integration) and in complex brain functions (memory, cognition, nociception, and attention). Hence, they are potential therapeutic targets for Alzheimer’s (α7, α4β2) and Parkinson’s diseases (α6, β5, α4β2), pain (α4β2, α9α10), and schizophrenia (α7) [65,66,67]. The hair cell α9α10 nAChR is a therapeutic target to prevent hearing loss and hyperacusis [68,69]. Presynaptic nAChRs mediate the release of several neurotransmitters, including dopamine, responsible for the rewarding and addictive properties of drugs [34,70]. Therefore, nAChRs are attractive targets for the management of drug abuse. The α4β2 nAChRs mediate, in part, the addictive properties of nicotine and have been targeted for smoking cessation therapies [64]. Although blocking receptor activation by antagonists is effective in reducing nicotine dependence, complete antagonism results in cravings and relapse. Therefore, a partial agonist that prevents nicotine from interacting with the nAChRs and at the same time maintains some of the nicotine-like reinforcement to relieve craving is sought [64,71]. nAChRs are also involved in non-neuronal cholinergic systems. The α3, α5, α7, α9, or β2 subtypes facilitate cell adhesion and proliferation in keratinocytes (for a review, see [72]), nAChR-mediated angiogenesis in endothelial cells [73,74], and cholinergic modulation of immune cells (for a review, see [75]). Therefore, nAChRs are also targets for anticancer drugs. Reduced growth and proliferation of breast and lung cancer cells have been achieved by inhibiting α9 and α7 receptors, respectively (for a review, see [76]). Nicotine and its metabolites are the leading carcinogens that initiate lung cancer [77]. These metabolites, along with nicotine, are potent agonists of the nAChRs, which established the link between nAChRs and the development and progress of lung cancer [78]. Activation of the α7 nAChRs enhances cell proliferation in lung cancer [79], whereas activation of the α3/α4-containing subtypes inhibits apoptosis [80]. Activation of these signaling mechanisms could be successfully terminated by antagonists of the α7, α3 nAChRs [80,81,82]. Further, genome-wide studies showed a strong correlation between the overexpression of the CHRNA5/A3/B4 genes encoding the α5, α3, β4 subunits and lung cancer [83]. Selective molecular probes, especially for the less explored α3β4 subtype, are required to study the physiological role of this subtype in disease progression. Similarly, blocking nAChRs has been used as a strategy to treat inflammatory diseases such as Crohn’s disease, epilepsy, rheumatoid arthritis, and ulcerative colitis (for a review, see [84]). For example, a silent agonist of α7 nAChRs exhibits antinociceptive and anti-inflammatory properties in animal models [85,86]. To achieve these goals, highly selective and potent agonists and antagonists are essential. Such agents also add to our understanding of the distribution and specific physiological roles of nAChR subtypes that are associated with cholinergic and non-cholinergic functions [87]. Selective probes also contribute to our understanding of the pharmacological profiles of the various nAChRs, their different stoichiometric arrangements, and their functional implications.

### 2.1. Classification of Three-Finger Neurotoxins

In the last two decades, a significant number of 3FTxs have been discovered with minimal functional characterization. In particular, regarding the effects of mutations (indels and substitutions) of the functional site residues in postsynaptic neurotoxins. Previously, we evaluated Type II neurotoxins (LNTXs) as having distinct structural differences, and therefore, we classified them further into three classes based on the number of C-terminal tail residues and variations in the functionally important residues [32]. Drysdalin, an antagonist of nAChR from Australian elapid *Drysdalia coronoides* venom, retains nanomolar affinity towards nAChRs despite the replacement of three functional site residues in loop II by non-conserved residues. These studies indicated the importance of functional rather than structural conservation of amino acid residues [32]. Our preliminary evaluation of neurotoxin sequences showed significant variations in functional site residues. Such variations may affect the subtype and interface selectivity towards various nAChRs. Identifying the differences and classification of these neurotoxins will provide impetus to functionally characterize them. Therefore, we have classified neurotoxins that belong to the 3FTx family (Table 1).

### 2.2. Type I (Short-Chain) Neurotoxins

As mentioned above, the functionally important residues in erabutoxin a that are involved in binding to *Torpedo* nAChR are Gln^7^, Ser^8^, Gln^10^, Lys^27^, Trp^29^, Asp^31^, Phe^32(36)^, Arg^33(37)^, Glu^38(42)^, and Lys^47(53)^ [27,88]. Mutation of residues Gln^7^ (to Leu, 23-fold), Ser^8^ (to Gly, 176-fold; to Thr, 780-fold), and Gln^10^ (Ala, 210-fold) in loop I led to significant loss of binding to nAChR. However, mutations Phe4Ala, Asn5Val, His6Ala, Ser9Gly, Pro11Asn, Gln12Ala, Thr13Val, Thr14Ala, Lys15Ala, and Thr16Ala did not cause significant loss (<8.8-fold) of binding. Similarly, deletion of Ser18 or mutation of Glu21Ala did not affect binding. In loop II, mutation of residues Lys^27^ (to Glu, 175-fold), Trp^29^ (to Phe, 67-fold), Asp^31^ (to His, 46-fold), Arg^33(37)^ (to Glu, 318-fold; to Gln, 187-fold; to Lys, 25-fold), and Glu^38(42)^ (to Leu, 25-fold) led to significant loss of binding. However, mutation of residues Tyr25Phe, Asn26His, Gln28Ala, Trp29His, Ser30Ala, Asp31Asn, Phe32(36)Leu, Gly34(38)Ser, Thr35(39)Ala, and Glu38(42)Lys did not cause significant loss (<8.6-fold) of binding. Interestingly, the Ile36(40)Arg mutation enhances the binding by 7-fold. Since several neurotoxins have positively charged Arg or Lys residues, these residues are also considered important for binding to nAChRs. In loop III, mutation of Lys47(53) to Ala led to a 32-fold loss of binding, but mutation of all other residues did not affect the binding. Most of these functional site residues are found on the concave side of erabutoxin. Thus, the nAChR-binding site is a discontinuous protein–protein interaction site with three short segments spread over each of the three loops (for details, see [88]). Among these, Lys^27^, Trp^29^, Arg^33(37),^ and Lys^47(53)^ are thought to constitute a “common functional core” and contribute to establishing conservative contacts with nAChRs [88].

Ackermann and Taylor studied the interaction between recombinant mouse muscle AChR subunits expressed in HEK cells and mutants of *Naja mossambica mossambica* toxin I [28]. Their results indicated that the two sites (α-γ and α-δ) can be differentiated by Lys^27^ and Arg^33(37)^ mutants [28]. Such discrimination of the two binding sites is less straightforward with the *Torpedo* receptor than with mammalian muscle-type AChRs [89]. Upon reanalysis, Antil et al. determined that some mutants of erabutoxin a, Ser8Thr, Gln10Ala, Lys27Glu, Asp31His, Arg33(37)Glu, and Lys47(53)Glu, could also discriminate the two binding sites [29]. For example, Asp31His leads to the loss of 1.6- and 284.3-fold, Ser8Thr leads to the loss of 10.7- and 1190-fold, Arg33(37)Glu leads to the loss of 11.8- and 690-fold, Gln10Ala leads to the loss of 13.3- and 522.8-fold, Lys47(53)Glu leads to the loss of 16.8- and 312.8-fold, and Lys27Glu leads to the loss of 31.4- and 522.8-fold in two binding sites. Although these mutants can differentiate between the two sites, it is unclear whether the drops in the affinity are towards α-γ or α-δ inter-subunit interfaces. Since the mutations of Lys27Glu and Arg33(37)Glu in *Naja mossambica mossambica* toxin I lead to greater loss of activity in binding to the α-γ interface [28], it is logical to assume that similar mutations in erabutoxin a also lead to greater loss of affinity at the α-γ interface. Interestingly, binding affinities of native and mutant toxins at the α-ε interface (found in the adult form of nAChR) are not yet known. Despite the caveats, these data suggest that the changes in the functional site residues affect the binding-site selectivity, receptor subtype selectivity, species specificity, and reversibility. These factors provided the impetus for finer classification of three-finger neurotoxins.

Previously, we showed that proline residues are commonly found in the flanking segments of contiguous protein–protein interaction sites [90]. We hypothesized that Pro residues play two important structural roles in the flanking segments: (a) they protect the integrity and conformation of the interaction sites by creating the barrier on either side of the protein–protein interaction sites by breaking the propagation of neighboring secondary structures; and (b) they help in the presentation of the protein–protein interaction sites by inducing kinks in the protein structures (for details, see [90]). Interestingly, two out of three segments of the erabutoxin functional sites contain proline residues. In loop I, Pro^11^ flanks one side of Gln^7^, Ser^8,^ and Gln^10^ residues, while in loop III, Pro^44(50)^ and Pro^48(54)^ flank both sides of Lys^47(53)^ residue. Thus, Pro residues appear to play an important structural role in discontinuous functional sites. As flanking Pro residues in the flanking segments of the functional residues enhance the binding/activity of the ligands to receptors [90], they were also considered in the classification.

**Figure 1:**
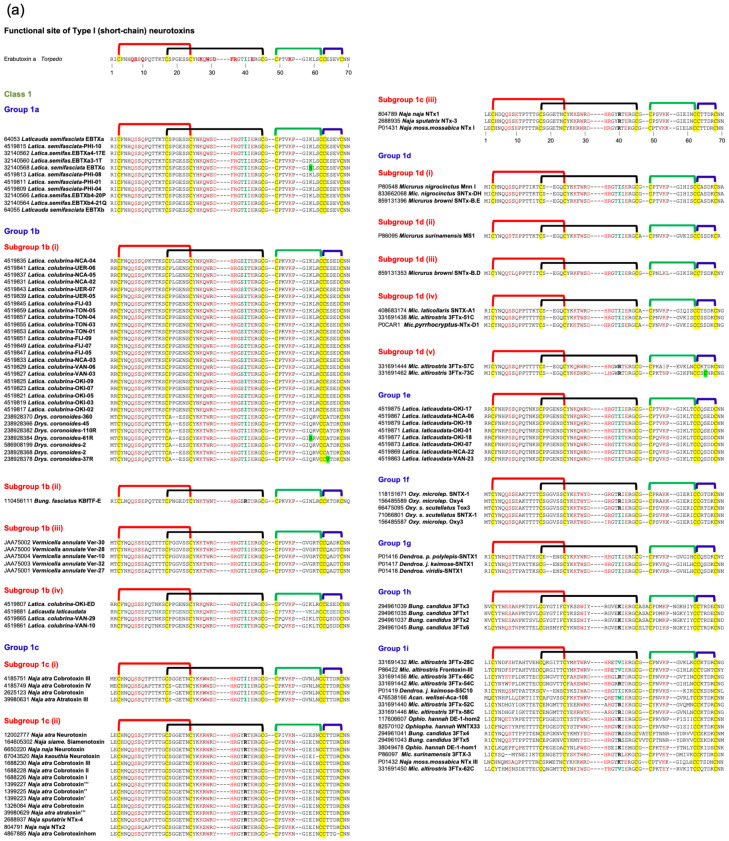

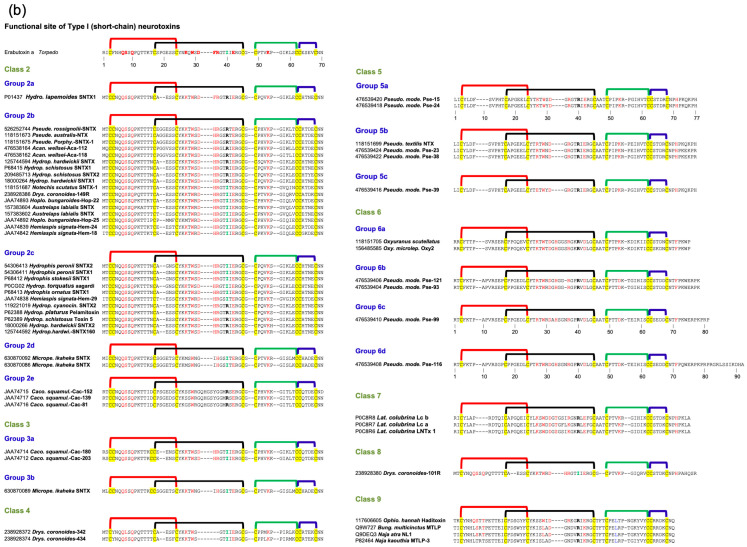
(**a**) Sequence alignment of Type I (short-chain) Class 1 neurotoxins. This class of neurotoxins is divided into nine groups. (**b**) Sequence alignment of Type I (short-chain) Class 2 to 9 neurotoxins. Functional residues, colored red, are based on mutagenesis studies on the binding of erabutoxin to the peripheral nAChR of the *Torpedo* electric ray. Erabutoxin, with Ile^36(40)^ (green), gained binding to the receptor when mutated to Arg^36(40)^ (black, bold). Sequences in this class of neurotoxins have a bulky, hydrophobic residue (green) or a positively charged residue (black, bold) at this position. Conserved disulfide-forming Cys are highlighted yellow. The four disulfide bonds are indicated above the sequences in red, black, green, and blue. The green highlight denotes an amino acid substitution with properties distinct from typical sequences in the group.

Type I neurotoxins are classified into nine classes based on the substitution of functional residues, the presence and location of Pro residues in the flanking segments, and C-terminal tail (Figure 1). Seven of these classes contain monomeric toxins, and one class has dimeric toxins, which are further divided into groups and subgroups.

#### 2.2.1. Type I Class 1

This class of neurotoxins is commonly found in elapid snake venoms. They are divided into nine groups. The group 1a neurotoxins contain erabutoxins and closely related neurotoxins. Thus, they contain all ten functional residues and the presence of Pro^11^, Pro^44(50),^ and Pro^48(54)^ residues flanking them. This group contains conserved Phe^32(36)^ and Ile^36(40)^. Most neurotoxins of the second group, 1b (i) resemble the first group except for the substitution of Phe^32(36)^ by His^32(36)^ residue (Figure 1a). The imidazole ring of His indeed can replace the phenyl ring of Phe to an extent in stacking interactions. In neurotoxins of group 1b (ii), uncharged Gln^10^ is replaced by negatively charged Glu^10^ residue. In one of the members, Asp^31^ and Ile^36(40)^ are replaced by Thr^31^ and Arg^36(40)^ residues, respectively. In group 1b (iii), Pro^11^ residue is missing, while in some group 1b (iv), Pro^48(54)^ residue is missing. The effects of these substitutions on the interaction of nAChRs are yet to be documented. The neurotoxins in the third group, 1c, have Pro^12^ instead of Pro^11^ residue (Figure 1a). Group 1c (i) neurotoxins have all three Pro residues: Pro^12^, Pro^44(50),^ and Pro^48(54)^. Neurotoxins in groups 1c (ii) and (iii) have only two Pro residues: Pro^12^ and Pro^44(50)^. These groups also have Ile^36(40)^ replaced by Arg^36(40)^ residue. In group 1c (iii) neurotoxins, Gln^10^ is replaced by negatively charged Glu^10^ residue (Figure 1a). All group 1d neurotoxins have Pro^11^ and Pro^12^ residues in loop I. Only the member of group 1d (ii) neurotoxin contains a negatively charged Glu^10^ residue that replaces the uncharged Gln^10^ found in group 1d (i) neurotoxins. In group 1d (iii) neurotoxins, there are two substitutions: Ser^8^ by Thr^8^ and Pro^48(54)^ by Leu^48(54)^. Groups 1d (iv) and 1d (v) neurotoxins have C-terminal Gly residues. Such Gly residues donate the amide group to the last but one residue, resulting in C-terminal amidation [91]. Post-translational C-terminal amidation is an important modification observed in about 50% of mammalian peptide hormones and more than 80% of insect hormones [91]. In vivo, a bifunctional enzyme, peptidylglycine α-amidating monooxygenase, catalyzes this reaction. Although C-terminal amidation has been observed in 3FTxs [92,93,94], it is not clear whether neurotoxins in these groups are amidated. There are two group 1d(v) neurotoxins, in which Lys^47(53)^ is replaced by Ile or Asn. In one, Lys^27^ is substituted by Gln^27^ residue, while in the other, Phe^32^ and Arg^33^ are substituted by Leu^32^ and His^33^ residues. All group 1e neurotoxins have Pro^7^ and Pro^11^ residues in loop I, one each on either side of Ser^8^ and Gln^10^ residues. A functionally important Gln^7^ residue is replaced by Pro^7^. Group 1f neurotoxins have only Pro^44(50)^ residue in loop III. These neurotoxins do not have Pro residues in loop I, and the Phe^32(36)^ is replaced by Gly^32(36)^ or Ile^32(36)^ residue (Figure 1a). In the three members of this group, Ile^36(40)^ is replaced by Arg^36(40)^ residue. In group 1g neurotoxins, Gln^10^ is substituted by Thr^10^ residue. In group 1h neurotoxins, only four functional residues, namely, Ser^8^, Trp^29^, Glu^38(42)^, and Lys^47(53)^, are retained, while Gln^10^ is substituted by conserved Asn^10^ residue in loop I, but all key functional residues in loop II and -III are conserved. Further, Ile^36(40)^ is replaced by Lys^36(40)^ residue. Group 1i neurotoxins have only some residues in each loop that are conserved. Thus, nAChR-binding activities of groups 1h and 1j neurotoxins are expected to be reduced significantly.

#### 2.2.2. Type I Class 2

This class of neurotoxins is commonly found in hydrophiid and Australian elapid snake venoms. They have a Cys^4^ residue, in addition to the conserved eight Cys residues (Figure 1b). This additional Cys is not involved in disulfide bond formation and remains as the free thiol [95,96]. They are subdivided into five groups. The first and second groups differ by one key functional residue: Phe^32(36)^ in group 2a and His^32(36)^ in group 2b. In group 2a and in some members of group 2b, Ile^36(40)^ is replaced by Arg^36(40)^ residue. In group 2c neurotoxins, Pro^48(54)^ is substituted by Ser, Thr, His, or Arg residue. In both group 2d neurotoxins, the four key substitutions in loop II functional residues are as follows: Lys^27^ by Met^27^, Asp^31^ by Gly^31^, Phe^32(36)^ by Ile^32(36)^, and Arg^33(37)^ by His^33(37)^. Group 2e polypeptides have an unusually longer, Gly/Ser-rich loop II than all Type I neurotoxins. Only Trp^29^ appears to be conserved, but there is a His residue in this segment. Because of the flexibility of Gly/Ser-rich segments, the exact topological position of these residues in either free or bound form is difficult to predict.

#### 2.2.3. Type I Class 3

This class of neurotoxins is found in *Cacophis squamulosus* (Australia) and *Micropechis ikaheca* (New Guinea) venoms. In addition to the Cys^4^ residue of Class 2 neurotoxins, they also have a Cys^16^ residue. Thus, they have ten Cys residues. It is unclear whether these additional Cys residues form the fifth disulfide in loop I or alter canonical disulfide pairings and the 3FTx scaffold. There are two groups: group 3a has all ten functional residues conserved, while group 3b has only seven (three out of six residues in loop II) functional residues conserved (Figure 1b).

#### 2.2.4. Type I Class 4

These four classes are atypical Type I neurotoxins. In Class 4 neurotoxins, three key functional residues in loop II, namely Asp^31^, Phe/His^32(36),^ and Arg^33(37)^, are deleted. They have Pro–Pro residues on either side of the Lys^47(53)^ residue (Figure 1b).

#### 2.2.5. Type I Class 5 to 8

Neurotoxin Classes 5, 6, 7, and 8 are structural intermediates between Type I and Type II neurotoxins. Loop I is relatively short in Type II neurotoxins, and hence, this loop is not involved in its interaction with nAChRs [29,30]. Instead, Phe^65(70)^ or His^65(70)^ (α-cobratoxin numbering) in the C-terminal extension interacts with nAChRs. Neurotoxins from classes 5, 6, and 7 have shorter loop I, and the typical functional residues, such as Gln^7^, Ser^8,^ and Gln^10^, responsible for binding to nAChRs, are absent. They have C-terminal extensions and His (Classes 5 and 7) or Phe (Class 6) residues (typical of α-bungarotoxin or α-cobratoxin, respectively), which are similar to Type II neurotoxins. In Class 5 neurotoxins, loop II has all functional residues, except for the Phe/His^32(36)^ residue. Pro residues flank both sides of Lys^47(53)^ in loop III and the His^65(70)^-equivalent residue in the C-terminal tail. Neurotoxins in this class can be divided into three groups based on the presence of Lys^27^, Arg^27^, or Glu^27^ (Figure 1b). Although loop II neurotoxins in classes 6 and 7 are as long as Type II neurotoxins, they do not have the characteristic fifth disulfide bond. In Class 6 neurotoxins, loop II has all three functionally equivalent residues, Asp^31^, His^32(36)^, and Arg^33(37)^, but there are Gly/Ser residues interspersing them. As mentioned above, these Gly/Ser residues may provide flexibility to the tip of loop II and help in binding to nAChRs. Neurotoxins in this class can be divided into four groups based on the length (7, 10, 13, and 24 residues, respectively) and sequence of the C-terminal tails. In Class 7 neurotoxins, His^32(36)^ is replaced by Thr/Ser^32(36)^ residues. Similarly to Class 5 neurotoxins, Pro residues flank both sides of Lys^47(53)^ in loop III and His^65(70)^-equivalent residue in the seven-residue C-terminal tail. Interestingly, the only member of Class 8 has all loop I and loop II characteristics of Type I neurotoxins but has a C-terminal extension with His residue, similar to Type II neurotoxins.

#### 2.2.6. Type I Class 9

Haditoxin from king cobra venom [97] is the first well-characterized member of this class of neurotoxins. It has functionally important Gln^7^, Ser^8^, Trp^29^, Asp^31^, Arg^33(37)^, Arg^36(40)^, and Glu^38(42)^ residues. It is a novel non-covalent homodimer of Type I neurotoxin, and it blocks muscle (αβγδ) and neuronal (α7, α3β2, and α4β2) nAChRs. This is the first dimeric neurotoxin that interacts with α7-nAChRs as well as with muscle nAChRs. Although haditoxin shows 75–80% sequence identity with 3FTxs [97], only a few functional residues of α-neurotoxins are conserved. The details of the dimer formation and dimer interface in 3FTxs have been described earlier [97]. Interestingly, both [Phe^23^ and Leu^48(54)^] residues involved in hydrophobic interactions and most of the residues involved in hydrogen bond interactions in dimerization are conserved (Figure 1b). Thus, this class of 3FTxs is most likely to form dimers. The members other than haditoxin have several unconserved substitutions of the functional residues [Gln7Leu, Trp29Leu, Arg33(37)Asn, and Glu38(42)Lys].

#### 2.2.7. Subgroups in Type I Neurotoxins

Class 1 has eighteen subgroups, with groups 1b, 1c, and 1d having four, three, and five subgroups, respectively, whereas all other groups have only one subgroup each. Classes 2, 3, 5, and 6 have five, two, three, and four groups, while other classes have one group each. Thus, there are 36 subgroups in Type I neurotoxins that differ from each other. The common functional core residues Lys^27^, Trp^29^, Arg^33(37)^, and Lys^47(53)^ are highly conserved (83.5–98.8%). Lys^27^, the least invariant (83.5%), is substituted by charged (Arg^27^, seven times; Glu^27^, four times), hydrophilic (Ser^27^, six times; Thr^27^, two times; Asn/Gln^27^, once each), and hydrophobic (Met^27^, five times; Leu^27^, once) or Gly^27^ (once) residues. Trp^29^ is the most invariant functional residue, with 98.8%, and it is replaced only twice by Phe^29^, an aromatic hydrophobic residue in monomeric neurotoxins. Arg^33(37)^ (94.7% conserved) is substituted by His^33(37)^ or Gly^33(37)^ (four times each) or Ser^33(37)^ (once). Lys^47(53)^ (93.0% conserved) is substituted by negatively charged (Asp^47(53)^, four times; Glu^47(53)^, twice), hydrophilic (Asn^47(53)^, five times), and hydrophobic (Ile^47(53)^, once) residues. Other functionally important residues, such as Gln^7^, Ser^8^, Gln^10^, Asp^31^, Phe^32(36)^, and Glu^38(42)^ residues that are conserved in Type I neurotoxins but not in Type II neurotoxins were identified as “functionally variant” residues [88]. These functional variant residues were proposed to be responsible for the species selectivity and subtype selectivity and these residues are also fairly conserved (Gln^7^, 84.1%; Ser^8^, 86.0%; Gln^10^, 69.6%; Asp^31^, 89.4%; His^32(36)^/Phe^32(36)^, 73.5%/7.6%; and Glu^38(42)^, 88.9%) in all the Type I neurotoxins. Thus, the variations in the functional site residues, the presence or absence of flanking Pro residues, and characteristics of C-terminal residues may contribute to differences in the subtype selectivity, subunit interface selectivity, species specificity, binding reversibility, and affinity towards nAChRs (or other Cys-loop receptors) of these subgroups of Type I neurotoxins.

### 2.3. Type II (Long-Chain) Neurotoxins

**Figure 2:**
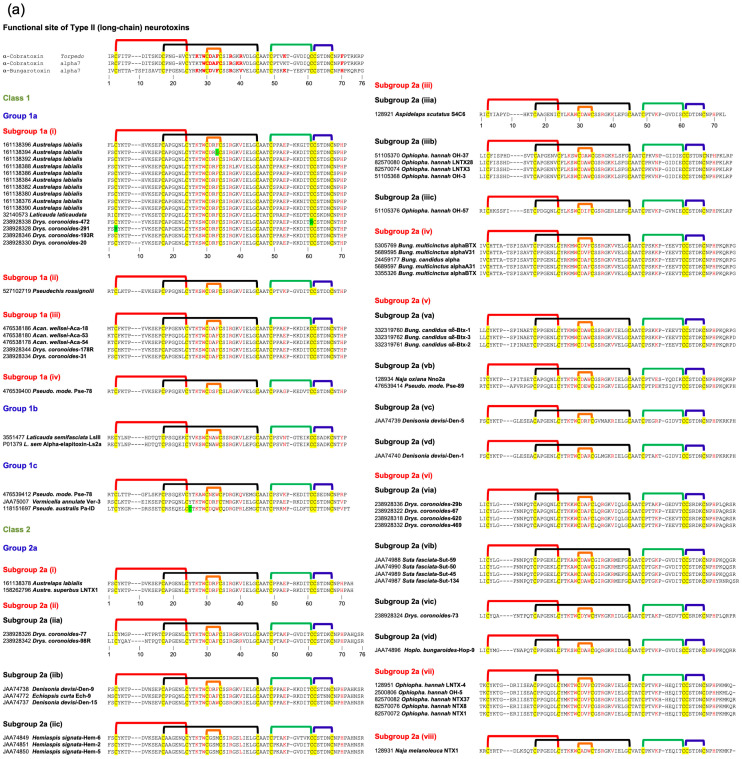

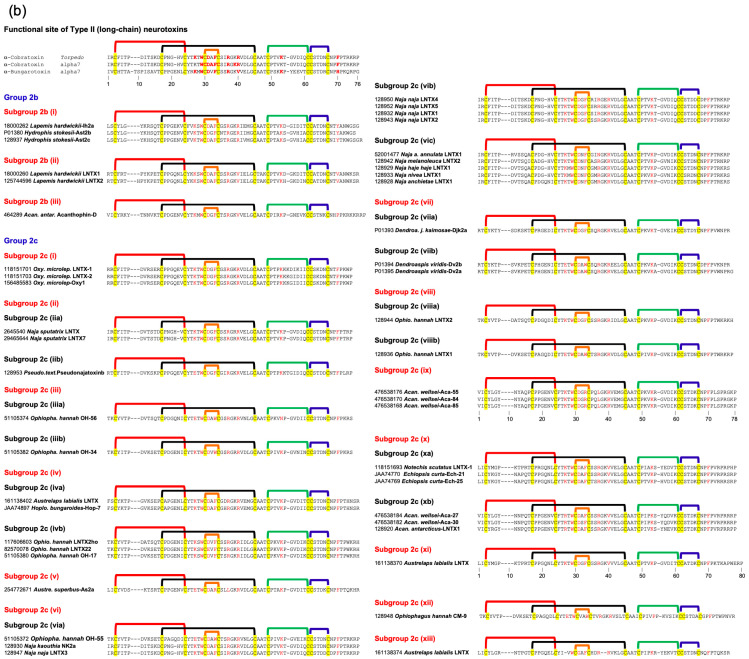

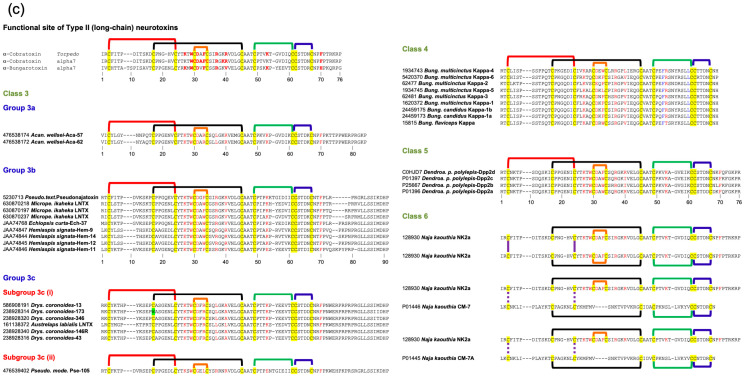
(**a**) Sequence alignment of Type II (long-chain) Class 1 and Class 2, Group 2a neurotoxins. (**b**) Sequence alignment of Type II (long-chain) Class 2, Groups 2b and 2c neurotoxins. (**c**) Sequence alignment of Type II (long-chain) Classes 3 to 6 neurotoxins. Functional residues, colored red, are based on mutagenesis studies on the binding of α-cobratoxin to the peripheral nAChR of the *Torpedo* electric ray. Conserved disulfide-forming Cys are highlighted yellow. The five disulfide bonds are indicated above the sequences in red, black, green, blue, and orange. In Class 6 sequences, intermolecular disulfides were indicated by purple line, with dashed line indicating inferred disulfides. The green highlight denotes an amino acid substitution with properties distinct from typical sequences in the group.

Both Type I and Type II neurotoxins bind to acetylcholine-binding pockets in the α-γ (or α-ε) and α-δ interfaces and block peripheral muscle-type nAChRs. Their binding affinities to both these sites are indistinguishable, and no cooperativity was observed [29]. In addition, Type II neurotoxins also block several neuronal nAChRs, such as α7, α9, and α9α10 [6]. Structurally, they differ from Type I neurotoxins by the presence of the fifth disulfide bond at the tip of loop II. The residues in α-cobratoxin that play an important role in binding to peripheral nAChR of *Torpedo* were identified by site-directed mutagenesis [29]. Interestingly, none of the loop I residues in α-cobratoxin contributes to binding, in contrast to Type I neurotoxins. Site-directed mutation of all 10 residues in loop I showed no significant effect on binding to *Torpedo* receptors [29]. Like Type I neurotoxins, loop II residues located on the concave side, namely, Lys^23(27)^, Trp^25(29)^, Asp^27(31)^, Phe^29(33)^, Arg^33(37)^, and Arg^36(40)^ (α-cobratoxin numbering), play a critical role in the binding. Structurally, these residues are in topologically comparable positions with Type I neurotoxins. The Lys23(27)Glu mutant distinguishes the two binding sites with a loss of 2- and 25.7-fold affinity. Mutation of Trp^25(29)^ to Phe^25(29)^ or His^25(29)^ does lead to 2- to 4-fold loss, while mutation to Ala^25(29)^ leads to 11-fold affinity [29]. Neutralization of the negative charge of Asp^27(31)^ in Asn^27(31)^ leads to minimal loss, but change to positively charged Arg^27(31)^ leads to 31-fold loss. Mutation of Phe^29(33)^ to Leu^29(33)^ leads to a 12.4-fold loss of activity. Arg^33(37)^ appears to be the most critical residue, and its mutation to Glu^33(37)^ leads to a whopping 767-fold loss of activity. The mutation of Arg^36(40)^ to Ala^36(40)^ leads to a 7.2-fold loss. Interestingly, the removal of the fifth disulfide in Cys26(30)Ser/Cys30(34)Ser double mutant does not affect the activity. Mutation of Lys^49(53)^ to Glu^49(53)^ in loop III leads to a loss of 3.1- and 52.7-fold activity. In the C-terminal tail, mutation of Phe65(70)Ala leads to a 6.8-fold loss. Thus, eight amino acid residues (Figure 2) form the complete functional site to bind to *Torpedo* nAChR [29].

By using systematic site-directed mutation of 34 residues in all three loops of α-cobratoxin, the binding site for the neuronal α7 receptor was delineated [30]. As in the case of binding to *Torpedo* nAChR, the mutation of loop I residues showed no significant loss of activity. Among loop II residues, mutation of Trp^25(29)^ (to Ala^25(29)^, 6-fold), Asp^27(31)^ (to Arg^27(31)^, 11-fold), Ala^28(32)^ (to Gly^28(32)^, 5.4-fold), Phe^29(33)^ (to Ala^29(33)^, 74-fold), Arg^33(37)^ (to Glu^33(37)^, 339-fold), Lys^35(39)^ (to Ala^35(39)^, 11-fold), and Arg^36(40)^ (to Ala^36(40)^, 16-fold) results in loss of activity towards α7 receptor. The mutation of three residues, including the mutation of Lys^49(53)^ to Glu^49(53)^, showed no loss of binding. The mutation of Phe^65(70)^ to Ala^65(70)^ shows a 15.5-fold loss of activity. The removal of the fifth disulfide [in Cys26(30)Ser/Cys30(34)Ser double mutant] leads to 13.9-fold loss of activity. Interestingly, there are two key differences in the functional site residues that are involved in the interaction of α-cobratoxin with *Torpedo* and α7 receptors. Lys^23(27)^ or Lys^49(53)^ play an important role in binding to the former, while the fifth disulfide bond appears to play a critical role in binding to the latter receptor.

Recently, we classified monomeric Type II neurotoxins into three classes [32]. Here, Type II neurotoxins are re-classified into six classes based on the substitution of functional residues, the presence of flanking Pro residues, and C-terminal tail (Figure 2); the first three classes contain monomeric toxins and are further classified into groups and subgroups, while the last three classes contain dimeric forms. Further, these monomeric dimeric Type II neurotoxins are further classified into groups and subgroups.

#### 2.3.1. Type II Class 1

This class of Type II neurotoxins is most commonly found in elapid and hydrophiid snake venoms. They have the shortest C-terminal tail (4–5 residues) and are divided into three subclasses. All Group 1a neurotoxins contain Val^36(40)^ and His^65(70)^ residues, which are similar to α-bungarotoxin (Figure 2a). The neurotoxins in this group are divided into four subgroups based on key residues in position 28(32) and 49(53). Group (i) and Group (ii) neurotoxins contain Arg^28(32)^ replacing the Ala^28(32)^ residue, but they differ by the presence of Glu^49(53)^ in group (i) and Lys^49(53)^ in Group (ii). Group (iii) neurotoxins contain Ala^28(32)^ and Glu^49(53)^, while a single group (iv) neurotoxin has Ser^28(32)^ and Gly^49(53)^ residues. Class 1b neurotoxins have four key residues, namely, Asp^27(31)^ by Asn^27(31)^, Phe^29(33)^ by Trp^29(33)^, Lys^49(53)^ replaced by Asn^49(53)^, and His^65(70)^ by Tyr^65(70)^ compared to Class 1a neurotoxins. All three members in Class 2c differ from each other in residues in positions 27(31), 28(32), 29(33), 36(40), 49(53), and 65(70), but all of them have a short C-terminal tail. With additional sequences, they may be further classified into distinct groups.

#### 2.3.2. Type II Class 2

This class of neurotoxins has slightly longer C-terminal tails (6–13 residues) and is divided into three subclasses (Figure 2). Group 2a neurotoxins (Figure 2a) have His^65(70)^ residue and are classified into eight groups. Group (i) neurotoxins have six residues in the C-terminal tail. Both neurotoxins have Arg^28(32)^, Phe^29(33)^, Val^36(40)^, Glu^49(53)^, and His^65(70)^ residues. Group (ii) neurotoxins have nine residues in the C-terminal tail with conserved NPHPAHXSR, where X is N, Q, or K residue. They are categorized into three subgroups based on the residues 27–29 (31–33), 36(40), and 49(53). Subgroup (iia) neurotoxins have Asp^27(31)^, Ala^28(32)^, Phe^29(33)^, Arg^36(40)^, and Lys^49(53)^ residues. In contrast, subgroup (iib) neurotoxins have Asp^27(31)^, Arg^28(32)^, Phe^29(33)^/Trp^29(33)^, Val^36(40)^/Arg^36(40)^, and Glu^49(53)^ residues, while (iic) neurotoxins have Gly^27(31)^, Ser^28(32)^, Met^29(33)^, Leu^36(40)^, and Lys^49(53)^ residues. The neurotoxins in group (iii) have 6–8 residues but with a distinct C-terminal sequence compared to Group (i) and (ii) neurotoxins. They are classified into three subgroups that differ from each other in residues at 29(33), 36(40), and 49(53) positions. A single toxin in subgroup (iiia) has Trp^29(33)^, Lys^36(40)^, and Lys^49(53)^ residues. Subgroup (iiib) neurotoxins have Trp^29(33)^, Lys^36(40)^, and Asn^49(53)^ residues, while (iiic) neurotoxins have Phe^29(33)^, Arg^36(40)^, and Lys^49(53)^ residues. Group (iv) neurotoxins are classical α-bungarotoxins isolated only from the genus *Bungarus* (krait) snakes. Key differences in the functional sites between α-bungarotoxin and α-cobratoxin are the presence of Val^36(40)^ compared to Arg^36(40)^ and His^29(33)^ compared to Phe^29(33)^ residues. α-Bungarotoxins have two distinct isoforms with Ala^28(32)^ and Val^28(32)^, but the impact of this mutation is not clear. Although Gly is the C-terminal residue, amidation in α-bungarotoxin is not yet reported. Neurotoxins in groups (v) through (viii) have 8–10 residues but differ from each other with distinct C-terminal sequences. Group (v) neurotoxins are further classified into four subgroups based on the residues in 29(33), 33(37), 36(40), and 49(53) positions. Neurotoxins in subgroups (va) and (vb) have Trp^29(33)^, Arg^33(37)^, and Val^36(40)^ residues but differ by the presence of Lys^49(53)^ (va) or Glu^49(53)^ (vb) residues. Neurotoxins in subgroups (vc) and (vd) have Met^33(37)^ and Arg^36(40)^ residues but differ by the presence of Phe^29(33)^ and Arg^49(53)^ (vc) or Arg^29(33),^ and Lys^49(53)^ (vd) residues. Group (v) neurotoxins are further classified into four subgroups based on the residues 28(32), 29(33), 33(37), and 36(40). Subgroup (via) neurotoxins have Ala^28(32)^, Phe^29(33)^, Arg^33(37)^, and Val^36(40)^ residues; subgroup (vib) neurotoxins have Ala^28(32)^, Trp^29(33)^, Arg^33(37)^ and Arg^36(40)^ residues; subgroup (vic) neurotoxins have Tyr^28(32)^, Trp^29(33)^, Lys^33(37)^ and Arg^36(40)^ residues; and subgroup (vid) neurotoxins have Ala^28(32)^, His^29(33)^, Arg^33(37)^ and Arg^36(40)^ residues. Group (vii) neurotoxins have all functional site residues conserved and are similar to α-bungarotoxins in group (iv), except for the differences in the loop I and C-terminal sequences. A single neurotoxin in group (viii) has Ala^27(31)^, Asp^28(32)^, and Trp^29(33)^, distinct from all other groups.

Group 2b neurotoxins (Figure 2b) have non-His^65(70)^/Phe^65(70)^ residues and are classified into three groups depending on residue 65(70). They have Ala^28(32)^/Gly^28(32)^ and Arg^36(40)^/Val^36(40)^ residues. Group (i) neurotoxins have Tyr^65(70)^ residue, and both *Hydrophis* toxins are amidated at the C-terminal [92]. Based on the presence of C-terminal Gly in *Lapemis hardwickii* neurotoxin, it is logical to assume that this protein is also amidated. Thus, this group is one of the few 3FTxs that are amidated at the C-terminal. Group (ii) neurotoxins have Val^65^ residue, while group (iii) neurotoxins have Pro^65(70)^ residue.

Group 2c neurotoxins (Figure 2b) have a Phe^65(70)^ residue, and the C-terminal has 7–13 residues. They are classified into 13 groups depending on the C-terminal sequences and the functional site residues. Groups (i)–(iii) neurotoxins have short C-terminals with only seven residues, each with a distinct C-terminal sequence. Groups (i) and (ii) neurotoxins have Gly^28(32)^ residue. Subgroup (iia) neurotoxins have Lys^23(27)^ residues, while the single subgroup (iib) neurotoxin has Glu^23(27)^ residues. Similarly, a single member of subgroup (iiia) has Glu^23(27)^, Ala^28(32)^, and Asn^49(53)^ residues, while subgroup (iiib) has Lys^23(27)^, Val^28(32)^, and Lys^49(53)^ residues. Groups (iv)–(vii) neurotoxins have nine C-terminal residues. In addition to differences in loop I and C-terminal sequences, group (iv) neurotoxins differ by the presence of Arg^36(40)^ and Phe^65(70)^ residues from α-bungarotoxins in Class 1a, group (iv). Subgroup (iva) neurotoxins have Ala^28(32)^, while (ivb) neurotoxins have Val^28(32)^ residues. Group (v) neurotoxin has Glu^23(27)^, Arg^29(33)^, and Leu^33(37)^ residues. Group (vi) neurotoxins are classified into three subgroups based on the residues in position 28(32); Ala^28(32)^ in (via), Gly^28(32)^ in (vib), and Asn^28(32)^ in (vic) neurotoxins. α-Cobratoxin belongs to group (via). Group (vii) neurotoxins have three members; one has a C-terminal Gly residue, while the other two are one residue short and probably have amidated C-terminals. All three toxins were sequenced by protein chemistry [98,99,100], before mass spectrometry was in use in determining the mass of proteins and peptides. Indeed, one of these toxins (P01395) shows the presence of C-terminal Gly [100], and hence, it is not amidated. The sequence of subgroup (viia) neurotoxins has Lys^23(27)^, Gly^28(32)^, and Phe^29(33)^ residues and subgroup (viib) neurotoxins have Glu^23(27)^, Ala^28(32)^, and Trp^29(33)^ residues. Group (viii) neurotoxins have 10 C-terminal residues and subgroup (viiia) neurotoxins have Lys^23(27)^, Gly^28(32)^, and Phe^29(33)^ residues, while subgroup (viib) neurotoxins have Glu^23(27)^, Ala^28(32)^, and Trp^29(33)^ residues. Neurotoxins in groups (ix) and (x) have 11 C-terminal residues. Group (ix) neurotoxins have Gly^28(32)^, Arg^29(33)^, Leu^33(37)^, and Arg^36(40)^ residues. Group (x) neurotoxins have Ala^28(32)^/Ser^28(32)^, Phe^29(33)^, Arg^33(37)^, and Val^36(40)^ residues. Group (xi) neurotoxin has 13 C-terminal residues. Interestingly, this neurotoxin does not bind to the neuronal α7 receptor [101]. Group (xii) neurotoxin has 10 C-terminal residues and differs from other neurotoxins by the presence of Val^27(31)^ and Pro^49(53)^ residues while group (xiii) neurotoxin has nine C-terminal residues, and several residues [27(31), 34(38), and 35(39)] are deleted in loop II. Further, Asp^27(31)^ is replaced by Gly^27(31)^ residue.

#### 2.3.3. Type II Class 3

This class of neurotoxins has the longest C-terminus (17–24 residues) and was divided into three subclasses (Figure 2c) [32]. Class 3a neurotoxins have 17 residues in the C-terminal tail. Classes 3b and 3c neurotoxins share similar C-terminal sequences. Class 3c neurotoxins have an additional 1–6 residues (mostly PR residues) inserted upstream of the homologous C-terminal region. Class 3c neurotoxins can be further classified into two groups. Class 3b neurotoxins have typical aromatic Trp^29(33)^/Phe^29(33)^ and positively charged Arg^33(37)^ residues, while in class 3a and group 3c (i) neurotoxins, these residues are replaced by Arg^29(33)^ and Leu^33(37)^ residues. In addition, in group 3c (i) neurotoxins, positively charged Arg^36(40)^ residue is replaced by Ala^36(40)^/Leu^36(40)^ residues. In group 3c (i) neurotoxin has Gly^27(31)^, Leu^29(33)^, and Glu^49(53)^ residues. Drysdalin (LNTX-13 from *Drysdalia coronoides* venom) lacks three of the conserved classical functional residues (due to Arg^29(33)^, Leu^33(37)^, and Ala^36(40)^ unconserved residue substitutions replacing aromatic and two Arg residues, respectively) involved in the interaction with nAChRs. Despite these drastic changes, it shows irreversible, postsynaptic neurotoxicity with nanomolar potency. It selectively inhibits the rodent muscle (α1)2β1εδ and hα7 and hα9α10 nAChRs, but not human α3β2, α3β4, α4β2, and α4β4 nAChRs. The reversal to conserved Phe^29(33)^ substantially leads to reduction or abolition of inhibitory activity, whereas reversal to Arg residue has minimal impact on the potency. The removal of the C-terminal 20 residues leads to complete loss in activity at α9α10 nAChR and irreversibility with reduced potency at the muscle and α7 nAChRs [32]. Although α-bungarotoxin and α-cobratoxin share similar nAChR selectivity with drysdalin, their binding to acetylcholine-binding proteins from *Lymnaea stagnalis* (Ls-AChBP) and *Aplysia californica* (Ac-AChBP) is significantly different; α-bungarotoxin and α-cobratoxin preferentially bind to Ls-AChBP, while drysdalin preferentially binds to Ac-AChBP [102]. Thus, the binding modalities of these long-chain neurotoxins to nAChRs and AChBPs are different.

#### 2.3.4. Type II Class 4

This class contains all the κ-neurotoxins isolated from *Bungarus* venoms. They block neuronal nAChRs, while the α-neurotoxins block nerve transmission at the muscle endplate. This group of toxins was first identified based on the blockade of neuronal nAChRs in the chick ciliary ganglion, which were not susceptible to α-bungarotoxin [8]. Such κ-neurotoxin-sensitive nAChRs are localized to postsynaptic sites in the ciliary ganglion [103]. κ-Neurotoxins most potently block α3-containing nAChRs (IC_50_ < 150 nM) but fail to block α2- and/or α4-containing very high-affinity nicotinic receptors (for details, see [8]). They bind to muscle-type nAChR with low affinity (*K*_d_ ~ 10 μM) and fast reversibility [104]. Unlike Type-II α-neurotoxins, κ-bungarotoxin exhibits lower or no affinity towards α7-, α8-, and α9-containing nAChRs. κ-Neurotoxins are homodimeric toxins formed by non-covalent interactions between two Type-II neurotoxins, unlike α-neurotoxins that are monomeric. The details of the dimer formation and dimer interface in 3FTxs are described below. Briefly, the residues involved in the dimerization are mostly conserved (Figure 2c). Thus, this class of 3FTxs is most likely to form dimers. Most of the functionally important residues, such as Lys^23(27)^, Asp^27(31)^, Phe^29(33)^, Arg^33(37)^, and Val^36(40)^ are conserved. However, Trp^25(29)^ is replaced mostly by Gln residue. Loop III is involved in dimerization, and Arg^49(53)^ replaces Lys^49(53)^. Unlike other Type II neurotoxins, this class of toxins has a short C-terminal tail, and thus, Phe^65(70)^ is also missing.

#### 2.3.5. Type II Class 5

This class contains toxins isolated from *Dendroaspis polylepis polylepis* (black mamba) venom. Among them, DPP2d is a well-characterized toxin [94]. This toxin is amidated at the C-terminus, a rare posttranslational modification in 3FTxs. Interestingly, this protein crystallizes as a dimer similar to κ-neurotoxins [105] and haditoxin [97]. This non-covalent homodimer is formed via a network of van der Waals interactions, four main chain–main chain hydrogen bonds, two main chain–side chain hydrogen bonds, and two interlocked salt bridges [Glu^54(58)^–Lys^56(60)^]. However, in solution at 150 mM of NaCl, it elutes as a monomer [94]. Salt at isosmotic concentrations appears to break the salt bridges at the dimeric interface. DPP2d blocks human muscle-type (α1β1γδ) and neuronal α7 nAChRs but not α3-containing nAChRs. Thus, its pharmacological profile resembles typical Type II α-neurotoxins and not κ-neurotoxins. This group includes three other *Dendroaspis* toxins, which were sequenced by protein chemistry in the 1970s when protein/peptide mass spectrometry had not been developed yet. Therefore, it is possible that they are also amidated at the C-termini. This group of toxins has a longer C-terminus, similar to Type II α-neurotoxins. The functionally important residues, such as Lys^23(27)^, Trp^25(29)^, Asp^27(31)^, Trp^29(33)^/Phe^29(33)^, Arg^33(37)^, Arg^36(40)^/Ile^36(40)^, Lys^49(53),^ and Phe^65(70)^, are conserved.

#### 2.3.6. Type II Class 6

This class contains disulfide-linked covalent homomeric and heteromeric dimers. They were isolated from *Naja kaouthia* venom [106]. The homodimer of α-cobratoxin (αCT-αCT) blocks *Torpedo* and α7 nAChRs but with lower affinity compared to the α-cobratoxin monomer. Interestingly, this dimer gains the ability to inhibit α3β2 nAChR similar to κ-neurotoxins. αCT-αCT is more active on Torpedo nAChR, but it is the least active dimer on α7 nAChR compared to heterodimers. Although heterodimers block *Torpedo* and α7 nAChRs, they lose the cytotoxicity [106]. Despite the presence of only one nAChR-binding loop II, the heterodimer blocks α3β2 nAChR better than αCT-αCT [107]. The crystal structure of αCT-αCT [107] indicates that two cysteine residues involved in the first disulfide bond, namely C1 and C3, are involved in the formation of two inter-subunit disulfide bonds (C1-C3′ and C3-C1′) (Figure 2c). The structures of the other two heterodimers are not yet determined. Most likely, they may have similar disulfide arrangements in the dimers. This class can be divided into two groups: group 6a with one homodimer αCT-αCT and group 6b with heterodimers.

#### 2.3.7. Subgroups in Type II Neurotoxins

Class 1 has three groups with six subgroups; group 1a has four subgroups, while all other groups have only one subgroup each. Class 2 has three groups with 42 subgroups; class 2c and 2a have 21 and 18 subgroups, respectively, while class 2b has only 3 subgroups. Class 3 has three groups and four subgroups, and group 3c has two subgroups. Class 6 has two groups, while the other classes have one group each. Thus, there are 56 subgroups in Type II neurotoxins that differ from each other. The common functional core residues Lys^23(27)^, Trp^25(29)^, Asp^27(31)^, and Arg^33(37)^ are highly conserved (88.7–94.7%). The other four functionally important residues, Phe^29(33)^, Val^36(40)^, Lys^49(53),^ and His^65(70)^/Phe^65(70)^, are poorly conserved (44–68%). Lys^23^ (88.7% conserved) is substituted by Arg^23(27)^ (4%) or Glu^23(27)^ (6.7%), and thus, this residue is mostly charged (positive charge, 92.7% and negative charge, 6.6%). Trp^25(29)^ is fully conserved in all monomeric Type II neurotoxins but is replaced by either Gln (5 times) or Arg/Ser/Leu (one time each) residues in dimeric κ-neurotoxins (Figure 2). Asp^27(31)^ (92% conserved) is replaced by Gly^27(31)^ (5 times), Asn^27(31)^ (thrice), Glu^27(31)^ (twice), or Val^27(31)^ (once) residues. Arg^33(37)^ (89.3% conserved) is substituted by hydrophobic (Leu^33(37)^, 12 times or Met^33(37)^, twice) or hydrophilic (Lys^33(37)^ or Asn^33(37)^, once each) residues. Phe^29(33)^ (61.3% conserved) is replaced by conserved aromatic Trp^29(33)^ (38 times, 25.3%), positively charged Arg^29(33)^ (14 times, 9.3%) or hydrophobic (Leu^29(33)^ or Met^29(33)^, thrice each) residues. As we showed recently in the case of drysdalin, replacement of the conserved aromatic residue by Arg^29(33)^ is probably tolerated as the interaction with nAChRs shifts from π-π to π-cation interaction [32]. Hydrophobic Val^36(40)^ (61.3% conserved) is mostly substituted by hydrophilic, charged Arg^36(40)^ (61 times, 40.7%) or Lys^36(40)^ (5 times, 3.3%) residues. It is also substituted by hydrophobic (Leu^36(40)^ or Ala^36(40)^, 5 times each or Ile^36(40)^, twice) residues. Lys^49(53)^ (68% conserved) is replaced by positively charged Arg^49(53)^ (9 times, 6%) or negatively charged Glu^49(53)^ (30 times, 20%) or uncharged Asn^49(53)^ (6 times, 4%) residues. Interestingly, Arg^49(53)^ is conserved in all eight κ-neurotoxins (Figure 2). His^65(70)^/Phe^65(70)^ (44% and 43.3% conserved, respectively) is substituted by aromatic Tyr^65(70)^ (5 times) or hydrophobic Val^65(70)^ (4 times) residues. Because of the shorter C-terminal tail, this residue is absent in κ-neurotoxins (Figure 2). Functionally important residues in loop I of Type I neurotoxins, such as Gln^7^, Ser^8^, and Gln^10^, are missing in Type II neurotoxins due to shorter loop I [88].

### 2.4. Type III Neurotoxins

Gong et al. isolated a new class of neurotoxins from *Pseudonaja textilis* venom [9]. They also sequenced six cDNAs encoding related isoforms. These neurotoxins were recombinantly expressed and characterized for their toxicities and binding to *Torpedo californica* nAChR. These toxins belonged to the 3FTx family but exhibited several unique features. They had only 57–58 amino acid residues; three loops are four, one, and three residues shorter, respectively, compared to erabutoxin a. The two native toxins isolated from the venom showed toxicity with 840 μg/kg and 800 μg/kg LD_50_ values in mice and inhibited the binding of [^125^I]α-bungarotoxin to nAChR with 2.6 × 10^−6^ and 2.3 × 10^−7^ M [9]. Six recombinant toxins showed 1 mg/kg LD_50_ values, and IC_50_ values ranged between 1.8 × 10^−7^ and 3.1 × 10^−6^ M. These values showed that their activities were weaker than those of typical Type I (short-chain) neurotoxins (LD_50_ range, 50–150 μg/kg; IC_50_ range, 10^−10^–10^−11^ M). We identified this new group as Type III neurotoxins [7]. None of the functional site residues that are involved in binding to nAChRs in Type I and Type II neurotoxins are conserved in this group of neurotoxins. The amino acid residues that are involved in the recognition and binding to nAChRs are yet to be determined.

**Figure 3:**
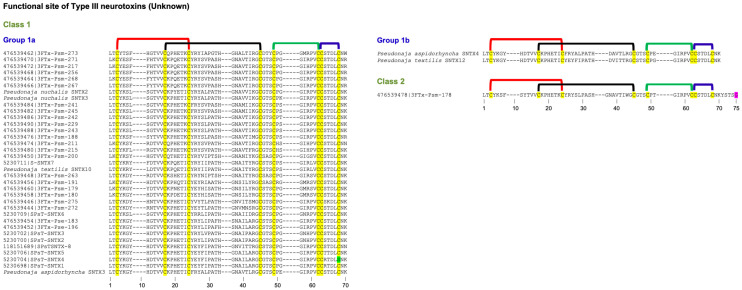
Sequence alignment of Type III neurotoxins. Functional residues are not known. Potential amidation for Gly at C-terminal in the lone sequence in Class 2 is highlighted in magenta. Conserved disulfide-forming Cys are highlighted yellow. The four disulfide bonds are indicated above the sequences in red, black, green, and blue. The green highlight denotes an amino acid substitution with properties distinct from typical sequences in the group.

This group of neurotoxins has been classified into Classes 1 and 2 (all from *Pseudonaja* genus). Class 1 is divided into two groups, which differ in the presence (Group 1a, 58 residues) or absence (Group 1b, 57 residues) of Gly^30(37)^ residue (Figure 3). Class 2 has an extended C-terminal tail with an additional five residues, including Tyr^59(71)^ corresponding to aromatic Phe^65^ in Type II neurotoxins.

#### Subgroups in Type III Neurotoxins

Class 1 has two groups, while Class 2 has one member group. Thus, there are three subgroups in Type III neurotoxins that differ from each other. Once the functional sites of these neurotoxins are delineated, they probably could be further classified.

### 2.5. Non-Conventional Neurotoxins

**Figure 4:**
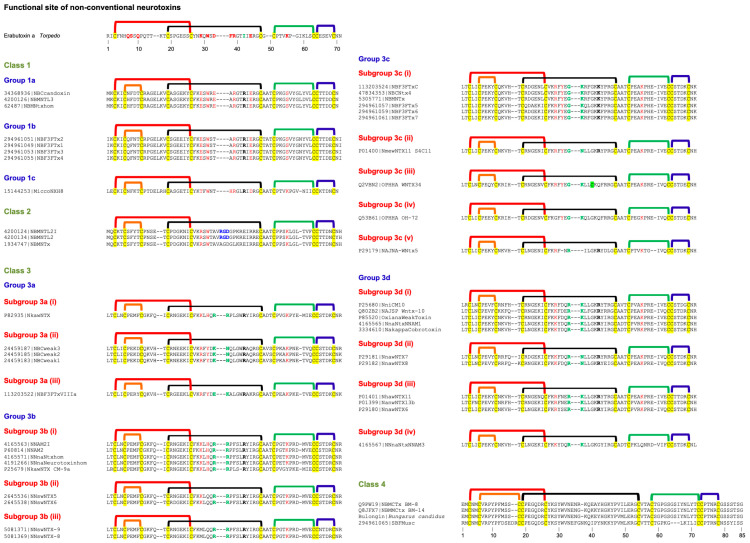
Sequence alignment of non-conventional neurotoxins. Compared to erabutoxin, functional residues that are conserved are colored red. Based on mutagenesis of WTX (Class 3) binding to nAchRs, Arg^31(33)^ and Arg^32(37)^ residues, colored green, are important functional residues. A positively charged residue at position 42 (bold, black) is potentially a specific determinant for interaction with mAChRs but not nAChRs. The integrin-binding tripeptide RGD in Class 2 sequences is colored blue. Conserved disulfide-forming Cys are highlighted yellow. The four disulfide bonds are indicated above the sequences in red, black, green, and blue.

These neurotoxins have five disulfide bridges: four of which are conserved among all 3FTxs, and the fifth disulfide is in loop I, unlike long-chain α-neurotoxins and κ-neurotoxins, which have the fifth disulfide in loop II (Figure 4). This class of toxins was first isolated from *Naja melanoleuca* venom [108] and hence, initially referred to as melanoleuca-type [109,110] or miscellaneous-type toxins [111]. Because of their lower order of toxicity (LD_50_ from 5 to 80 mg/kg) as opposed to prototype α-neurotoxins (LD_50_, 0.04–0.3 mg/kg), they were referred to as weak toxins [10,11]. However, the lethality of these toxins is notoriously variable; for example, the LD_50_ of γ-bungarotoxin (*B. multicinctus*) is 0.15 mg/kg [112], which is comparable to that of α-neurotoxins. Considering the distinct disulfide pairing, this large group of toxins was classified as non-conventional toxins [14,113]. Such toxins were first isolated from elapid venoms but subsequently were also found in colubrid [114,115] and viperid [116] venoms. As with other 3FTxs, non-conventional toxins do not belong to a functionally homogeneous class of toxins. Here, we will focus on non-conventional neurotoxins that block nAChRs. They are classified into four classes based on similarity of the functional site residues with Type I and Type II neurotoxins and the pharmacological profiles.

#### 2.5.1. Non-Conventional Neurotoxins Class 1

This class of neurotoxins was classified as Groups II and IV Orphan toxins [7]. They have most of the loop II functional residues that are found in Type I neurotoxins. Some of them also have Lys^47(55)^ in loop III, but none of them have equivalent functional residues in loop I. Further, due to the presence of the fifth disulfide bridge, this loop is turned away or out of plane with the rest of the structure [117,118]. However, there is no direct evidence for the participation of these residues in binding to nAChRs. They have been classified into three groups. Group 1a has six loop II functional residues: Glu^27(29)^, Trp^29(31)^, Glu^31(33)^, Arg^33(39)^, Arg^36(42)^, and Glu^38(44)^. Asp^31(33)^ is replaced by conserved, negatively charged Glu^31(33)^ residue. Positively charged Lys^47(55)^ in loop III is replaced by Ser^47(55)^ residue. Candoxin belongs to this group, and it binds to and blocks rat (α1)2β1γδ muscle-type nAChR with an IC_50_ of 10 nM [13]. Although IC_50_ values are comparable with Type I and II neurotoxins, candoxin shows reversible binding in contrast to the irreversible binding of the classical neurotoxins. Interestingly, candoxin irreversibly blocks α7 nAChR with an IC_50_ of 50 nM despite the lack of disulfide bridge in loop II of Type II neurotoxins [30]. Thus, other members of this group will also exhibit similar high-affinity binding to muscle-type and α7 nAChRs. Group 1b differs from Group 1a neurotoxins by the presence of Thr^31(33)^ replacing negatively charged Glu^31(33)^ residue. The only member of Group 1c has only Trp^29(31)^, His^32(38)^, Arg^33(39)^, Arg^36(42)^, Glu^38(44)^ and Lys^47(55)^ residues that are found in Type I neurotoxins.

#### 2.5.2. Non-Conventional Neurotoxins Class 2

This class is represented by γ-bungarotoxin [112]. This class of neurotoxins was classified as Group V Orphan toxins [7]. Although this group of neurotoxins has only Arg^27(29)^, Trp^29(31)^, and Lys^47(55)^ residues that are found in Type I neurotoxins, γ-bungarotoxin exhibited high toxicity and showed postsynaptic effects. It binds to both *Torpedo california* nAChR and M2 muscarinic AChR (mAChR) with IC_50_s of ~180 nM and ~150 nM, respectively [119]. In addition, it also inhibits platelet aggregation in platelet-rich plasma with an IC_50_ of ~30 nM due to the presence of the R^33(35)^GD^35(37)^ tripeptide sequence that plays a critical role in adhesive functions [120,121,122]. In one of the members of this group (#1934747), Arg^33(35)^ is replaced by Ala^33(35)^ residue, and hence, this toxin may not inhibit platelet aggregation. This is an unusual group of neurotoxins that binds to nAChR, mAChR, and integrin αIIbβ3 (with the exception of #1934747) with nanomolar affinity. Detailed structure–function relationships of this class of neurotoxins will be of great interest.

#### 2.5.3. Non-Conventional Neurotoxins Class 3

This class of neurotoxins is the most studied non-conventional toxin. WTX from *Naja kaouthia* venom was the first to be characterized for its interaction with nAChRs [10,11]. It blocks the binding of α-bungarotoxin to *Torpedo californica* nAChR with an IC_50_ of ~2.2 μM and inhibits ACh-induced current of rat α7 and human α7 with IC_50_s of 8.3 μM and 15 μM, respectively [10,11]. Because of the low affinity, it was named as “weak toxin” [10,11]. We, on the other hand, thought that this group of toxins may have a distinct target other than nAChRs and classified them as Group II Orphan toxins [7]. So far, no other non-neurotoxic target for these toxins has been identified.

Most of the neurotoxins in this group have Arg^27(29)^/Lys^27(29)^, Tyr^29(31)^/His^29(31)^ and Lys^47(55)^ residues that are found in Type I neurotoxins. The structure–function relationships of loop II residues of WTX from *Naja kaouthia* venom have shown the importance of basic residues Arg^31(33)^ and Arg^32(37)^ in binding to nAChRs (highlighted in green, Figure 4) [123]. The substitution of these residues by Ala leads to a 3-5-fold loss of binding affinity towards *T. californica* and α7 nAChRs. When both residues are replaced, the mutants fail to bind to α7 nAChR [123]. The Arg31(33)Ala/Arg32(37)Ala mutant completely lost its activity on M1, M2, and M3 subtypes of human mAChRs [124]. The mutation of Arg^37(42)^ to Ala had no significant effect on binding to nAChRs but led to complete loss of activity against all mAChR subtypes [123,124]. Thus, the Arg^37(42)^ residue appears to be a specific determinant required for the interaction of WTX with mAChRs but not with nAChRs. The substitution of W^36(41)^ by Ala residue leads to a ~2-fold reduction in affinity toward *T. californica* but not towards α7 nAChRs [123]. Similarly, this mutation leads to a reduction in the activity on M2 but not on M1 and M3 mAChRs [124]. Keeping all these functional residues in view, these neurotoxins were classified into four groups.

Group 3a toxins have Trp^36(41)^, similar to the first well-characterized toxin from *Naja kaouthia* venom, which was named tryptophan-containing neurotoxin [11]. They are further classified into three subgroups based on the residues in 27(29), 29(31), 31(33) and 32(38) positions. The subgroup 3a (i) toxin has Lys^27(29)^, His^29(31)^, Arg^31(33)^, and Arg^32(37)^ residues. Both subgroups 3a (ii) and 3a (iii) toxins have Arg^27(29)^ and Tyr^29(31)^ but differ by the presence of Lys^31(33)^ and Asn^32(37)^ residues in subgroup 3a (ii) toxins and Glu^31(33)^ and Lys^32(37)^ residues in subgroup 3a (ii) toxins. Group 3b toxins have Leu^36(49)^, and they are further classified into three subgroups based on the residues in the 27(29), 29(31), 31(33), and 32(37) positions. The subgroup 3b (i) toxins have Lys^27(29)^, His^29(31)^, Arg^31(33)^, and Arg^32(37)^ residues. In CM-9a from *Naja kaouthia* venom Leu^36(41)^ is deleted and Lys^47(55)^ is replaced by Tyr^47(55)^ residue. Thus, these toxins resemble subgroup 3a (i) toxin [11] except for the Trp^36(41)^ and the impact of this substitution is not clear. In subgroup 3b (ii) toxin His^29(31)^ is replaced by Gln^29(31)^ residue, while in subgroup 3b (iii) toxins Lys^27(29)^ and His^29(31)^ are replaced by Met^27(29)^ and Gln^29(31)^ residues, respectively. Group 3b (ii) synthesized based on the wNTX5 sequence from *Naja sputatrix* venom gland inhibits binding of α-bungarotoxin to *Torpedo* nAChR with a *K*_d_ of 180 nM but exhibits poor affinity (90 μM) towards chicken α7 nAChR [12]. Groups 3c and 3d toxins contain Lys^36(41)^ residue. Group 3c toxins were identified as Orphan toxins II [7]. They can be further classified into five subgroups based on the residues in 27(29), 29(31), 31(33), 32(37) and 37(42) positions. Subgroup 3c (i) toxins have Arg^27(29)^, Tyr^29(31)^, Gly^31(33)^, Lys^32(37)^ and Lys^37(42)^ residues. The other four subgroups have one toxin each. Subgroup 3c (ii) toxin has Asn^32(37)^ and Arg^37(42)^ residues. Subgroup 3c (iii) and (iv) toxins have an uncharged Gln^37(42)^ residue instead of positively charged Lys/Arg residues. Subgroup 3c (iii) toxin has an additional Cys^35(40)^ residue, while in subgroup 3c (iv) toxin Arg^27(29)^ is replaced by Gly^27(29)^ residue. In subgroup 3c (v) the toxins have Arg^27(29)^, Arg^31(33)^ and Arg^37(42)^ residues but Tyr^29(31)^ and Lys^32(37)^ residues are deleted. In group 3d toxins can be further classified into four subgroups; Tyr^29(31)^ is replaced by negatively charged Asp^29(31)^ residue in subgroups 3d (i), (ii) and (iv) toxins, while it is replaced by uncharged Asn^29(31)^ or Ser^29(31)^ residue in subgroup 3d (iii) toxins. A single neurotoxin in subgroup 3d (iv) Arg^37(42)^ and Lys^47(55)^ are replaced by Gly^37(42)^ and Gln^47(55)^ residues, respectively. Oxiana weak toxin (P85520), a member of subgroup 3d (i), blocks rat α7 nAChR (IC_50_ = 2.2 μM) and *Torpedo* nAChR (IC_50_ = ~30 μM) [125].

#### 2.5.4. Non-Conventional Neurotoxins Class 4

This class of neurotoxins is much longer compared to other classes of non-conventional toxins and has a slightly altered location of the fifth disulfide bond in loop I. The disulfide linkages in this class of toxins are experimentally not established, but they may be similar to other non-conventional toxins (Figure 4). The deduced amino acid sequence of the first member of this group was identified as cardiotoxin-like protein from *Bungarus multicinctus* [126]. Subsequently, native proteins BM8 and BM14 were purified from the same venom [127]. BM8 showed the identical protein sequence as that of the deduced sequence [126], while Glu^37(40)^ and Ala^38(41)^ in BM8 were replaced by Lys residues in BM14 [127]. A similar toxin was isolated and sequenced by Edman degradation from *Bungarus candidus* (Indonesia) venom. This toxin shows substitutions in three positions [Lys38(41)Glu, Ile46(49)Met, and Thr56(59)Ser] compared to BM14 (R. M. Kini, unpublished observations). This toxin was named bulongin (*Bungarus* long toxin) because of its unusually long loops. These toxins were identified as group XVII orphan toxins [7]. Neither protein shows cytolytic activity of cardiotoxins. Only BM14 inhibits binding of [^3^H]quinuclidinyl benzilate to M2 but not M1 type of muscarinic receptor [127]. Modification of the Lys^37(40)^ and Lys^38(41)^ of BM14 with trinitrobenzene sulfonate abolished its M2-binding, suggesting the role for these Lys residues [127].

Interestingly, according to Chung et al., BM8 and BM14 failed to inhibit α-bungarotoxin binding to *Torpedo californica* nAChR up to 100 μM [127]. In contrast, Utkin et al. showed that BM8 inhibits α-bungarotoxin binding to *T. californica* nAChR (IC_50_, 31 nM) and human α7 nAChR (IC_50_, 43 nM) [128]. The reasons for such discrepant results are unclear. The functional site residues that are involved in binding to nAChRs are yet to be determined.

Another toxin from *Bungarus flaviceps* (294961065) also has longer loops [129]. Interestingly, all three loop tips are distinctly different from the Class 4 toxins described above. Therefore, most likely this toxin will exhibit different pharmacological profiles.

#### 2.5.5. Subgroups in Non-Conventional Neurotoxins

Class 1 has three groups. Classes 2 and 4 have one group each, while class 3 has four groups and fifteen subgroups. Thus, there are 20 subgroups in non-conventional neurotoxins. Several amino acid residues in the α-neurotoxin functional site are topologically conserved in some of the potent neurotoxins, while a reduction in the number of functional site residues appears to lower the potency. Two basic amino acid residues at the tip of loop II have also been identified to contribute to their interaction with nAChRs and mAChRs [123,124].

### 2.6. Ω-Neurotoxins

**Figure 5:**
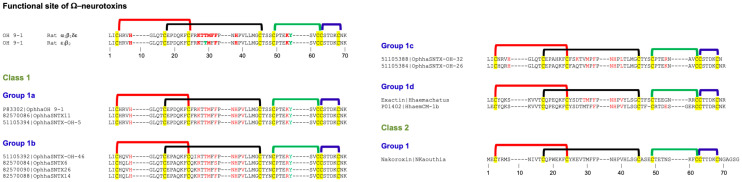
Sequence alignment of Ω-neurotoxins. Functional residues, colored red, are based on mutagenesis studies for the binding of Oh9-1 to rat α1β1εδ. Mutation of Thr^24(29)^ and Tyr^46(54)^, colored green, leads to increased inhibition against rat α3β2 nAChR. Conserved disulfide-forming Cys are highlighted yellow. The four disulfide bonds are indicated above the sequences in red, black, green, and blue.

These neurotoxins, similar to Type III neurotoxins, have 57 amino acid residues and four conserved disulfide bonds and bind to nAChRs with affinities in the micromolar range. Loops I and III are shorter compared to all neurotoxins. Oh9-1 from *Ophiophagus hannah* venom is the first member to be characterized [119]. It irreversibly inhibits carbachol-induced muscle contraction with an IC_50_ of 88 nM (only 4-fold less than that of α-bungarotoxin). Our studies showed that Oh9-1 blocks only rat peripheral α1β1γδ (fetal) and α1β1εδ (adult) and rat neuronal α3β2 nAChRs but not neuronal α7, α9α10, α4β2, and α4β4 nAChRs and any other human Cys-loop receptors, such as GABA_A_ (α1β2γ2 and ρ1 subtypes) and glycine (GlyRα1) receptors [15]. Indeed, Oh9-1 competes with ACh to bind to nAChR, similar to classical Type I and II neurotoxins. Interestingly, it lacked most of the functional site residues found in Type II (short-chain) α-neurotoxins [27,88]. Therefore, we evaluated the role of twelve residues {one, nine, and two residues at the tip regions [except Pro^28(33)^ and Pro^31(39)^] of loops I, II, and III, respectively]} by site-directed mutation to Ala and binding studies to rat α1β1εδ and α3β2 nAChRs. Our data suggested that mutations of His^7^ (loop I), Lys^22(27)^, Thr^23(28)^, Thr^24(29)^, Met^25(30)^, Phe^26(31)^, Phe^27(32)^, His^30(38)^ (loop II), and Lys^45(53)^ (loop III) lead to loss of binding to rat α1β1εδ nAChR, and the Tyr46(54)Ala (loop III) mutation leads to increased inhibition [15] (Figure 5). Thus, these residues are involved in binding to α1β1εδ nAChR and form the functional site. Interestingly, mutations of His^7^, Lys^22(27)^, Met^25(30)^, Phe^27(32)^, His^30(38)^ (loop II) and Lys^45(53)^ (loop III) lead to loss of binding to rat α3β2 nAChR, while mutations of Thr^24(29)^ and Tyr^46(54)^ lead to increased inhibition [15]. Thus, unlike both surfaces of loop II being involved in binding to α1β1εδ nAChR, only one surface appears to be involved in binding to α3β2 nAChR [15]. Based on the functional residues, these neurotoxins are classified into four groups. Group 1a toxins contain all the defined functional site residues of Oh9-1. In Group 1b toxins, Lys^22(27)^ and Lys^45(53)^ are replaced by Arg residues. Despite the conserved, positively charged guanidine group or Arg may have a distinct contribution compared to the amino group of Lys. In Group 1c toxins, Thr^24(29)^ and Tyr^46(54)^ are changed to Val^24(29)^ and Asn^46(54)^, respectively. Mutation of these residues increased the affinities towards α1β1εδ and α3β2 nAChRs [15]. In Group 1d toxins, both loops I and II have several changes. In addition, CM-1b from *Hemachatus haemachatus* venom has changes in key loop II residues. Nakaroxin, the only member of Class 2, does not inhibit the ligand binding of α-bungarotoxin to *Torpedo* muscle-type and α7 (α-bungarotoxin) and α4β2 (epibatidine) receptors [130]. This toxin interestingly enhances α-bungarotoxin binding to Ls-AChBP. Oh9-1 inhibits rat adult muscle-type (αβεδ) and rat α3β2 nAChRs [15]. This discrepancy could be due to either species or subtype selectivity. Thus, these toxins may exhibit more drastic functional differences among α-neurotoxins.

#### Subgroups in Ω-Neurotoxins

Classes 1 and 2 have one and four groups, respectively. Thus, there are five subgroups in Ω-neurotoxins.

### 2.7. Σ-Neurotoxins

**Figure 6:**
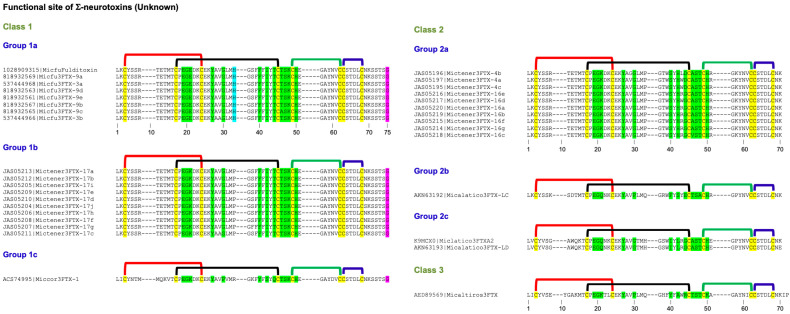
Sequence alignment of Σ-neurotoxins. Functional residues are not known. Fulditoxin is not amidated at C-terminal despite the presence of Gly (highlighted in magenta). Residues involved in dimerization are highlighted in green. Zn^2+^-binding His that facilitates tetramerization of dimers is highlighted in cyan. Conserved disulfide-forming Cys are highlighted yellow. The four disulfide bonds are indicated above the sequences in red, black, green, and blue.

We recently purified and characterized a new class of neurotoxin, fulditoxin, from the venom of the coral snake *Micrurus fulvius fulvius* [16]. It exhibits postsynaptic neuromuscular blockade of CBCM at nanomolar concentrations, comparable to Type I and II α-neurotoxins (IC_50_ values: fulditoxin, 27.8 nM; α-bungarotoxin, 11.4 nM; erabutoxin b, 31.7 nM). Interestingly, this neuromuscular blockade is completely reversible. Further, fulditoxin blocks cloned rodent (α1)_2_β1εδ and human (α1)_2_β1εδ nAChRs with IC_50_ values of 2.6 μM and 2.5 μM, respectively, about 100-fold lower affinity compared to avian muscle nAChR [16]. Unlike short-chain α-neurotoxins that bind only to muscle nAChRs, fulditoxin blocks cloned human neuronal α4β2, α7, and α3β2 nAChRs, which it blocked with IC_50_ values of 1.8, 7, and 12 μM, respectively. Fulditoxin does not have any of the functional site residues found in either canonical Type I (short-chain) α-neurotoxins [27,88] or Ω-neurotoxins [15]. Despite the absence of the key functional residues, fulditoxin binds to the ACh-binding site in nAChRs, similar to α-neurotoxins and Ω-neurotoxins [16]. The crystal structure of fulditoxin reveals its novel dimeric quaternary structure. It is a non-covalent dimer held together by hydrophobic interactions. It also binds Zn^2+^ ions and forms tetrameric complexes (for details, see [16]). These studies identified all the residues involved in dimer formation and the His residue at the tip of loop II that binds Zn^2+^ ions (Figure 6). Thus, dimerization in fulditoxin expands its pharmacological targets. However, the functional site residues and the pharmacophore that contribute to its interactions with nAChRs are not known. Fulditoxin shares 95–99% identity with seven *Micrurus* 3FTxs (Figure 6). Hence, we named this new class of nAChR-targeting neurotoxins Σ-neurotoxins. In addition, there are 25 other toxins that show high identity (>68%) and have retained most of the residues involved in dimerization. These *Micrurus* 3FTxs are likely to form non-covalent dimers, and hence, they may belong to the classification of Σ-neurotoxins.

Σ-neurotoxins are classified into three classes based on the C-terminal segment. Class 1 toxins have five additional C-terminal residues. Although they have Gly residue at the C-terminal end, at least fulditoxin is not amidated. It is unclear whether the C-terminals of other toxins are amidated or not. They are classified into three groups. Fulditoxin is the member of Group 1a. In Group 1a toxins, the amino acid residues involved in dimerization and the His residue involved in binding to Zn^2+^ and resulting in tetramerization of dimers are conserved (Figure 6). In Group 1b toxins Zn^2+^-binding His residue is replaced by Pro. In addition, Glu^17(21)^ is replaced by Lys^17(21)^ residues. In Group 1c toxins, His is replaced by Arg. Further, there are three unconserved substitutions compared to Group 1b toxins, Gly26(30)Pro, Ile35(42)Ser, and Thr37(44)Gln in the dimeric interface. Thus, Group 1b and 1c toxins do not form tetramers of dimers. However, fulditoxin data suggests that Zn^2+^-binding and tetramerization of dimers appear not to be important for neurotoxicity [16]. Class 2 neurotoxins have shorter C-terminals compared to Class 1 toxins. This is due to the insertion of T nucleotide from AGC (Ser) codon, leading to TAG stop codon in the first three out of four groups. Group 2a toxins are structurally similar to Group 1a toxins; most of the residues involved in dimerization are conserved. His residue is involved in Zn^2+^ binding, and tetramerization of dimers is also conserved (Figure 5). In Group 2b and 2c toxins, His residue is replaced by Pro and Gln residues, respectively. Some of the residues are replaced in these two groups. In the only member of Group 2d, there is a 12-nucleotide insertion with TGA to extend the C-terminal by two additional residues. Thus, the Group 2 toxins may form dimers but not tetramers of dimers. Class 3 neurotoxin has two additional residues at the C-terminal. This toxin probably forms a dimer similar to other Σ-neurotoxins.

#### Subgroups in Σ-Neurotoxins

Classes 1 and 2 have three groups each, while Class 3 has one group. Thus, there are seven subgroups in Σ-neurotoxins.

### 2.8. Colubrid Neurotoxins

Due to the smaller quantities of venoms [131], the studies on isolation and characterization of colubrid neurotoxins were about 4–5 decades delayed compared to those of elapid and viperid toxins. Although preliminary electrophoretic and enzymatic profiles of some colubrid venoms were available ([131,132,133,134,135] and references therein), we isolated the first postsynaptic neurotoxin, α-colubritoxin, from the Asian ratsnake *Coelognathus radiatus* (formerly, *Elaphae radiata*) [136]. It showed reversible neurotoxicity in chick biventer cervicis muscle (CBCM) preparations. Unlike elapid toxins, the N-terminal of α-colubritoxin is blocked by pyroglutamic acid and has an additional seven-residue segment at the N-terminal compared to elapid 3FTxs [136]. We also showed the presence of similar toxins in other colubrid venoms [136,137,138]. Subsequently, we purified and characterized denmotoxin from the mangrove catsnake (*Boiga dendrophila*) venom [114]. It exhibits species-specific postsynaptic neurotoxicity; it potently and irreversibly inhibits twitches in CBCM preparations. In contrast, it shows much weaker and readily reversible inhibition of twitches in mouse hemidiaphragm nerve-muscle preparations. The crystal structure of denmotoxin is similar to those of other 3FTxs [114]. The unique features of denmotoxin include the twisted tip of the central loop that originates from the kink of Pro^40(56)^, and the unusually long N-terminal segment is unstructured and appears to be flipping above the core. Not only are the canonical functional site residues of α-neurotoxins (except for Lys in loop III) and the typical concave binding surface missing, but the electrostatic potential of the tip of loop II is negative [114]. The full-length cDNA and the gene encoding the precursor of denmotoxin revealed that denmotoxin is the first 3FTx synthesized as a precursor protein, and the propeptide segment, encoded by the newly added exon (exon 2), is removed during maturation [139]. During maturation, the N-terminal Gln residue is also circularized, probably through venom glutaminyl cyclase [140]. We isolated and characterized a novel heterodimeric 3FTx, irditoxin, from the venom of brown treesnake *Boiga irregularis* [115]. The subunits in the dimer are covalently connected through an interchain disulfide bond formed between the unpaired Cys residues in loop I (B subunit) and loop II (A subunit). Both subunits are also produced as precursors, similar to denmotoxin. Irditoxin shows taxon-specific lethality toward birds and lizards and was nontoxic toward mice. It blocks the avian neuromuscular junction with comparable potency as α-bungarotoxin but blocks the mammalian neuromuscular junction 1000-fold less effectively [115].

Colubrid venoms and purified 3FTxs show drastic differences in their toxicity towards reptilian (e.g., birds and lizards) and mammalian (e.g., rodents, guinea pigs, and rabbits) (for details, see [141]). Therefore, structure–function relationships of colubrid toxins, in general, and nAChR antagonists, in particular, will contribute to our understanding of species-specific toxicity. Since the characterization of denmotoxin and irditoxin, amino acid sequences of several monomeric and dimeric 3FTxs have been determined by transcriptomic and proteomic studies of several colubrid Duvernoy’s glands and venoms, respectively [142,143,144,145,146,147,148]. All these toxins belong to non-conventional 3FTxs with the fifth disulfide bond in loop I [14]. A few of these colubrid toxins exhibit potent avian/reptilian-selective neurotoxicity [114,115,147,149]. The yet-to-be-determined functional site residues appear to be distinct from all the above classes of neurotoxins. As most functional residues are found in loop II of 3FTxs, based on a systematic analysis of amino acid sequences, this loop, conserved sequences, CYTLY, and WAVK are identified to play key roles in avian/reptilian-selective neurotoxicity [149]. Further, loop II is a bit longer in these toxins. However, specific amino acid residues, which are responsible for the interaction with nAChRs and species-specific neurotoxicity, are yet to be determined.

#### 2.8.1. Colubrid Neurotoxins Class 1

**Figure 7:**
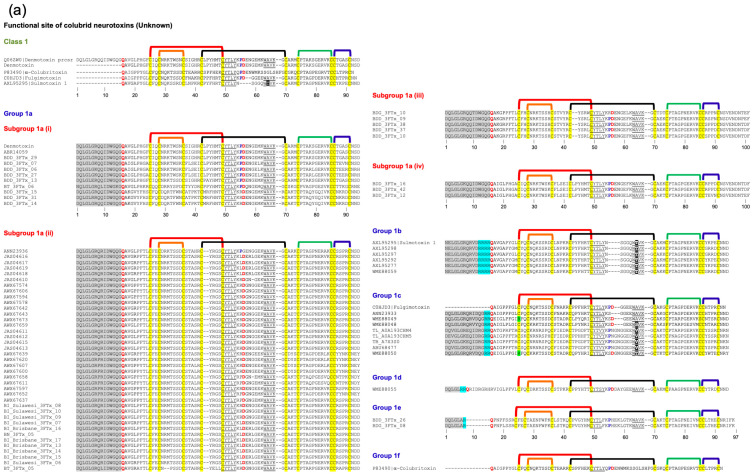

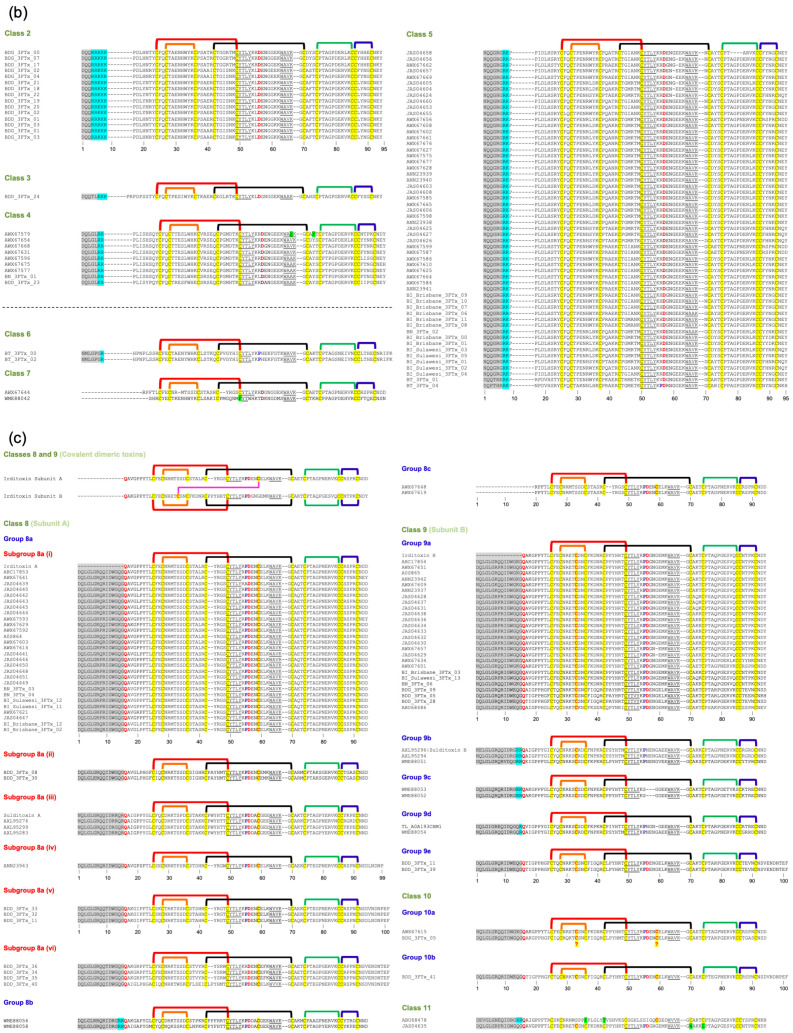
(**a**) Sequence alignment of colubrid neurotoxins, Class 1. (**b**) Sequence alignment of colubrid neurotoxins Classes 2 to 7. (**c**) Sequence alignment of colubrid neurotoxins Classes 8 to 11. Functional residues are not known. Propeptide region is shaded in gray, with potential basic processing sites highlighted in cyan. N-terminal Gln in Class 1 colubrid neurotoxins, colored red, are post-translationally modified to pyroglutamic acid. Conserved loop II residues CYTLY and WAVK are identified to play a key role in avian/reptilian-selective neurotoxicity (underlined). Non-Ala residues within the WAVK segment are highlighted in black. In Denmotoxin, Pro^40(55)^ (colored blue) caused a twisted tip of the central loop. The crucial positively charged loop II tip residue (typically Arg) in short- and long-chain α-neurotoxins is replaced by Asp^41(56)^ (colored red). This residue may bind to Arg193 of the δ-subunit to determine species selectivity. The green highlight denotes an amino acid substitution with properties distinct from typical sequences in the group. Conserved disulfide-forming Cys are highlighted yellow. The five disulfide bonds are indicated above the sequences in red, black, green, blue, and orange. Cys residues forming intermolecular disulfide (magenta line) in Classes 8 and 9 are colored red with yellow highlight.

The colubrid neurotoxins can be broadly classified as monomeric and dimeric toxins. The monomeric toxins are classified into seven classes based on the maturation sites and processes (Figure 7a). Upon maturation, all class 1 toxins have an N-terminal Gln that is most likely circularized to pyroglutamic acid, and a seven-residue segment extension compared to typical 3FTx. This class contains all five isolated toxins, namely denmotoxin, α-colubritoxin, boigatoxin A, fulgimotoxin, and sulmotoxin 1. This class of neurotoxins is classified into six groups. Denmotoxin is a well-characterized member of Group 1a toxins [114,139]. The precursor has a fifteen-residue propeptide segment, and the protease, which cleaves this segment, is yet to be identified. Proteases that cleave the Xaa-Gln peptide bond play a critical role in the maturation of bradykinin potentiating peptides [150,151]. Similar proteases are also important in cleavage and maturation of crotoxin, Mojave toxin, metalloproteases, and related toxins ([152] and references therein). Such proteases are found in crotalid venoms. It is possible that similar proteases are responsible for cleavage and removal of propeptide segments in Group 1a neurotoxins. Groups 1b and 1c neurotoxins have also fifteen-residue propeptide segments, but they have tetrabasic and dibasic processing sites, respectively, as was observed with peptide hormone processing sites [153,154] (Figure 7a). Group 1b neurotoxins, exemplified by sulmotoxin 1, have two back-to-back dibasic sites compared to one dibasic site in Group 1c. Furin or PACE (Paired basic Amino acid Cleaving Enzyme) and related enzymes in Golgi cleave at the dibasic sites [155,156,157]. Loop II of Group 1b neurotoxins is four residues shorter compared to Groups 1a and 1c neurotoxins and has WTVK, WSVK, and WVVK sequences instead of WAVK (Figure 7a). Groups 1d and 1e have dibasic and monobasic processing sites, respectively. The processing leaves a 17- and six-residue extension compared to typical 3FTxs. There is no data available on propeptide segment and the processing of Group 1f member, α-colubritoxin [136]. The functional impact of these changes is yet to be established.

#### 2.8.2. Colubrid Neurotoxins Class 2 to 7

Colubrid neurotoxins in Classes 2 (RRKKK), 3 (KKK), 4 (RR/KR), and 5 (RK) also have dibasic processing sites (Figure 7b). The mature toxins, however, have N-terminal Pro residues, unlike Gln in Class 1 neurotoxins. The processing rates with Pro residues in the P1′ site are slightly slower [158]. Class 2 toxins have eight-residue propeptides and five-residue N-terminal extensions, Class 3 toxins have eight-residue propeptides and seven-residue N-terminal extensions, Class 4 toxins have seven-residue propeptides and six-residue N-terminal extensions, and Class 5 toxins have eight-residue propeptides and seven-residue N-terminal extensions compared to canonical 3FTxs. In contrast, Class 6 toxins have a monobasic processing site, seven-residue propeptides, and six-residue N-terminal extensions (Figure 7b). Class 7 toxins do not have the propeptide segment and have only 3- and 1-residue N-terminal extensions. A careful evaluation indicates that exon 2 (of denmotoxin [139]) is missing in these toxins. Hence, no maturation occurs after signal peptide processing.

#### 2.8.3. Colubrid Neurotoxins Class 8 to 11

Based on the sequence similarity with the corresponding subunits of irditoxin and sulditoxin, Classes 8 and 9 toxins most likely form the A and B subunits of covalent heterodimeric toxins, respectively. They have extra Cys residues in loop II and loop I, respectively, that are involved in the formation of interchain disulfide bonds [115]. Class 8 (subunit A) toxins are classified into four groups based on the proteolytic maturation process. The processing of Group 8a toxins occurs by cleavage of the Xaa-Gln peptide bond, and they are classified into six subgroups. Toxins in Subgroups 8a(i) and 8a(iii) have dibasic processing sites on the N-terminal site of Gln residue, similar to those of A subunits of irditoxin and sulditoxin, respectively, and show subtle differences in loops I and II (Figure 7c). The members of Subgroups 8a(iv), 8a(v), and 8a(vi) have 5-, 7-, and seven-residue extensions, respectively, at the C-terminal end due to insertion of a single nucleotide (C) just ahead of the stop codons, leading to the frameshifts. In toxins in Groups 8b have dibasic processing sites, similar to Class 1, Group 1c toxins, with identical propeptide and N-terminal extensions. In Group 8c, both toxins do not have the propeptide segment due to missing exon 2, and hence, no maturation occurs after signal peptide processing.

Class 9 (subunit B) toxins are classified into five Groups based on the proteolytic cleavage in the maturation process. The processing of Group 9a toxins occurs by cleavage of the Xaa-Gln peptide bond, while that of Group 9b and 9c toxins occurs through the dibasic sites. Group 9d toxins have monobasic processing sites. Group 9e toxins have C-terminal extensions (Figure 7c). Class 10 toxins have both extra Cys residues in loops I and II that are found individually in subunits of heterodimers. They may form interchain disulfide bonds with other subunits, leading to the formation of novel quaternary structures. Or these two Cys residues may form an intrachain disulfide bond leading to the formation of unique monomeric toxins. Class 11 has two distinct toxins with two missing conserved Cys residues of the non-conventional toxins’ disulfide network. It is difficult to propose suitable disulfide linkages in both these examples.

All colubrid neurotoxins share conserved CYTLY and WAVK sequences in their loop II. In a small number of toxins, there are some conserved substitutions in these segments. These segments are proposed to be responsible for the avian/reptilian-selective neurotoxicity [149]. There are only a few exceptions. Only in Group 1b, toxin WAVK is replaced by WTVK; a hydrophobic residue is changed to a hydrophilic residue. In one of the Class 11 toxins, CYTLY is replaced by CGGHL. Further, the sequence of loop II in these toxins is also different (Figure 7c). The impact of these changes in the structure and function of the toxins will be of interest.

#### 2.8.4. Unique Gene Organization of Colubrid Toxins and Role of New Exon 2

**Figure 8:**
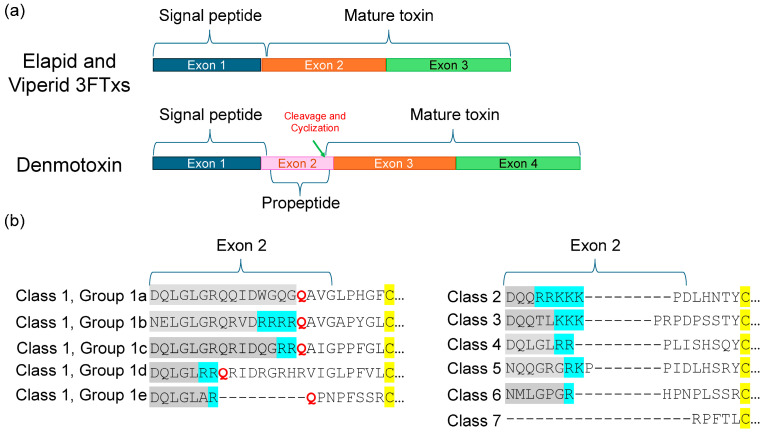
Gene organization of colubrid neurotoxins. (**a**) Compared to elapid and viperid 3FTxs, Class 1 colubrid neurotoxins, represented by denmotoxin, have an exon insertion (exon 2, pink) resulting in a propeptide segment in translated protein. (**b**) Exon 2 insertion can be observed in most classes of colubrid neurotoxins, resulting in at least 10 distinct propeptide segments. N-terminal Gln that may be modified to pyroglutamic acid is colored red. Propeptide region is shaded gray. Potential basic propeptide processing site are highlighted with cyan. Conserved disulfide-forming Cys are highlighted yellow.

The genes encoding 3FTxs in elapid (e.g., [159,160,161,162]) and viperid snakes [163] share identical organization, consisting of three exons and two introns (Figure 8a). Exon 1 encodes the 5′-untranslated region (UTR) and 18 amino acid residues of the signal peptide. Exon 2 encodes the last three amino acid residues of the signal peptide and the N-terminal half of the mature toxin, and exon 3 encodes the C-terminal half of the mature protein and the 3′-UTR. Exon 1 is conserved, while exons 2 and 3 are highly variable among various 3FTx genes. Elapid and viperid 3FTxs have two amino acid residues at the N-terminal of the first Cys residue. Interestingly, colubrid toxins (e.g., α-colubritoxin, denmotoxin, irditoxin subunits) are expressed as toxin precursors [139]. Their propeptide segments are cleaved off, and the newly exposed N-terminal Gln residue is cyclized to pyroglutamic acid [140]. Thus, mature colubrid toxins have additional seven-residue extensions compared to elapid and viperid 3FTxs. To understand the origin of this extension, we determined the denmotoxin gene organization [139]. The denmotoxin gene has four exons (Figure 8a); exon 1 encodes for 5′-UTR and most of the signal peptide, exon 2 encodes for propeptide and three amino acid residues of the mature toxin, exons 3 and 4 encode for sequences similar to elapid and viperid 3FTx genes. The signal peptide regions of elapid, viperid, and colubrid 3FTxs are highly conserved. Exons 2 and 3 of elapid and viperid 3FTx genes show accelerated evolution. Such accelerated evolution in toxins is due to an increased number of point mutations [164,165,166,167,168], accelerated segment switch in exons to alter targeting (ASSET) [163,169], rapid accumulation of variations in exposed residues (RAVERS) [170], and exonization and intronization [171]. Interestingly, colubrid neurotoxins show at least 10 distinct exon 2 segments that vary in their size and sequence (Figure 8b). These exons 2 contribute to propeptide segments, which, upon proteolytic processing, result in N-terminal extensions. A small number of the toxins do not appear to have propeptide segments. This could be due to either the absence of this exon 2 or alternate splicing. The unusually high number of distinct exons 2 may contribute to accelerated evolution. Currently, the impact of propeptide segments and their processing on the function of colubrid toxins is unclear.

#### 2.8.5. Subgroups in Colubrid Neurotoxins

Monomeric colubrid toxins have five classes. Class 1 has six groups with eight subgroups; group 1a has three subgroups, while all other groups have only one subgroup each. Classes 2 through 7 have six groups each, with one subgroup. Covalent heterodimeric toxins have 4 classes, 12 groups, and 17 subgroups. Thus, there are 31 subgroups in colubrid neurotoxins.

### 2.9. Endogenous and Exogenous Agonists, Antagonists, and Beyond

In vertebrates, nAChRs are formed by pentameric assembly of 17 homologous nAChR subunits (α1–α10, α1–α4, γ, δ, and ε) around a central ion-conducting pore. Only α subunits bind ACh through cysteines 192–193 located near the entrance to transmembrane helix 1; the other subunits lack these cysteines and thus, do not contribute to ACh-binding [34,172]. The peripheral muscle-type nAChR is formed by α1–γ/ε–α1–δ–α1 arrangement (anticlockwise when viewed from the extracellular space) (γ in fetal and ε in adult) with ACh-binding sites located at α1–γ/ε and α1–δ subunit interfaces of the extracellular domains. The neuronal nAChRs are either heteropentamers comprising diverse combinations of two or three types of subunits or homopentamers (α7 or α9) [34,173]. The ACh-binding sites in these nAChRs are located at α–β and α–α interfaces, respectively. The ACh-binding sites are formed by three highly conserved loops (A–C) of the principal (+) component α subunit and three less conserved loops (D–F) of the adjacent, complementary (–) component (mostly non-α subunit) [174,175]. The vicinal cysteines in loop C cover a hydrophobic agonist-binding pocket containing multiple aromatic residues of the binding loops. The ammonium group of ACh establishes a direct cation–π interaction with the tryptophan residue from loop B [176]. Consequently, the two components of the agonist-binding sites are nonequivalent, and the loops contribute differently to receptor function [177]. Thus, this distinct microenvironment at the ACh-binding interfaces defines affinities and selectivity towards various agonists and antagonists [173,178].

Although endogenous agonist ACh activates various subtypes of nAChRs, the ACh concentration threshold required to open the ion channel and the intensity of current generated vary significantly due to distinct binding interfaces. Thus, the selective activation of a specific nAChR is entirely regulated by in situ release of Ach, and the newly released ACh activates nearby nAChR to exhibit its function. Therefore, tightly controlled release of endogenous agonist ACh plays an important role in various physiological roles, and any alterations in their release and/or binding to relevant nAChR lead to neurological diseases. Further, the concentrations of ACh in the synaptic cleft depend on several factors including the number of vesicles, the location of release sites, the rate of diffusion, the geometry of the cleft and arrangement of active zones, the availability of binding sites on nAChRs, and rate of destruction by acetylcholinesterase (for details, see [179,180]). Exogenous agonist nicotine, like ACh, binds and indiscriminately affects the function of various nAChR subtypes based on the compartmentalization, accessibility, and binding affinity.

The binding of an agonist induces conformational changes and leads to the rotational torque in the β-barrel to rotate TM2 to open the cation channel [181]. Agonists must bind to two sites in heteromeric receptors or three sites at non-consecutive subunit interfaces in homomeric receptors in order to activate them [182]. In contrast, binding of an antagonist to one ACh-binding site is enough to block the activation. On the other hand, partial or silent agonists are molecules that produce little or no channel opening but selectively induce the desensitized state of nAChRs. Roger Papke and colleagues have designed several silent agonists [183,184,185,186,187]. The opened ion channels of nAChRs are targeted by pore blockers for various applications [188,189,190,191,192,193,194]. There are also positive and negative allosteric modulators designed to target various nAChRs for the treatment of several neurological disorders and diseases [68,195,196,197,198,199,200,201,202]. The efficacy of these ligands (or drug leads) depends on subtype and selectivity as well as their affinity, distribution, and accessibility. Such limitations provide great opportunities and intriguing challenges in designing highly specific, high-affinity ligands to several distinct regions of nAChRs to tailor-make the therapeutic leads.

### 2.10. Animal Toxins Inspire the Design of Highly Selective Ligands

Toxins can distinguish subunit interfaces, and hence, they will be useful in decoding highly variable subtypes and their interfaces [203,204,205]. α-Conotoxin MI binds to the α-δ interface with 30,000-times higher affinity than α-γ interface (*K*_D_, α-δ = 6.2 × 10^−10^ M and *K*_D_, α-γ = 2.0 × 10^−5^ M) [203]. Residues 93, 152–154, and 180–200 in α subunit form the principal site for ligand binding [206], while residues 34, 111, and 172 in γ subunit and corresponding residues in the δ subunit form the basis for subunit specificity; these residues are likely found at the interfaces of the two binding sites [207]. Waglerin-1 shows a 2000-fold preference for α-ε over the α-γ and α-δ interfaces [203]. Residues 57–61 and 172–174 in the ε subunit are responsible for the specificity of the waglerins [208,209]. Type I (short-chain) α-neurotoxin from *Naja mossambica mossambica* shows a 10,000-fold preference for the α-γ and α-δ interfaces over α-ε. Pro^175^ and Glu^176^ in γ subunit contribute to the high-affinity binding. These two residues are replaced by Thr^175^ and Ala^176^ in ε subunit [210]. As mentioned above, some of *Naja mossambica* toxin mutants differentiate α-γ and α-δ sites in mouse muscle nAChRs, while others do not [28,211]. Lys27Glu and Arg33(37)Glu mutations lead to 30- and 22-times more drop in binding affinities (*K*_D_) at the α-γ interface compared to α-δ interface. In contrast, Lys47(53)Ala mutation leads to an identical drop in binding affinities in both interfaces [211]. Similarly, Ser8Thr, Gln10Ala, Lys27Glu, Asp31His, Arg33(37)Glu, and Lys47(53)Glu mutants of erabutoxin a could discriminate α-γ and α-δ binding interfaces of *Torpedo* nAChR [29]. These authors also observed that several mutants of cobratoxin were able to discriminate α-γ and α-δ binding interfaces of *Torpedo* nAChR. These studies and many others indicate that changes in the functional site residues could affect the interface selectivity, receptor subtype selectivity, species specificity, and reversibility.

### 2.11. Subtle Variations in Conserved Functional Site Residues in α-Neurotoxins

**Figure 9:**
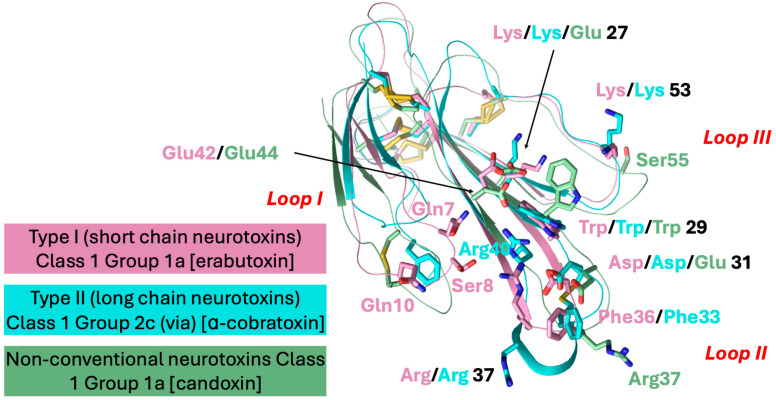
Structural alignment of Type I (pink), Type II (cyan), and non-conventional (green) neurotoxins showing functional residues, many of which are topologically conserved. Residues are numbered by homology numbering indicated in Figure 1, Figure 2, Figure 3, Figure 4, Figure 5, Figure 6 and Figure 7. PDB id for erabutoxin: 1QKD; α-cobratoxin: 7ULG; candoxin: 1JGK.

The conservation of functional residues is essential for retaining/maintaining selective binding to specific target receptors, ion channels, and/or enzymes. This is clearly visible in the case of α-neurotoxins. Short-chain (Type I; Figure 1) and long-chain (Type II; Figure 2) differ from each other in the loop sizes. In the long-chain neurotoxins, loop I is two to four residues short, whereas loops II and III are four or one/two residues longer, respectively. In short-chain neurotoxins, there are only two C-terminal residues after the last Cys, with only exception of hybrid neurotoxins. In contrast, long-chain neurotoxins have four to 24 C-terminal residues, with the exception of κ-neurotoxins, which have two residues. Further, there is a conserved fifth disulfide bond in loop II of long-chain neurotoxins. Despite these structural differences, they share common, topologically conserved amino acid residues that are involved in their interaction with nAChRs (Figure 9). They share common core residues consisting of Lys^27^/Lys^23(27)^, Trp^29^/Trp^25(29)^, Asp^31^/Asp^27(31)^, Arg^33(37)^/Arg^33(37),^ and Lys^47(53)^/Lys^49(53)^ to interact with different nAChRs [27,29]. These residues establish contact with invariant residues on the nAChRs. In addition, there are functional residues that help them bind selectively to muscle-type or neuronal α7 nAChRs. Based on subtle variations in these functional site residues, we have classified Type I and II neurotoxins into 36 and 56 subgroups, respectively. There is experimental evidence that even subtle, conserved substitutions lead to distinct subtype/interface selectivity. For example, native erabutoxin cannot distinguish αγ and αδ sites in *Torpedo* nAChR. In contrast, the Ser8Thr (conserved substitution) mutant shows 100-fold affinity differences between these sites [29]. Therefore, we expect functional differences among various subclasses. These functional site residues are also topologically conserved in non-conventional neurotoxins, such as candoxin (Figure 9). Such differences could be in interface selectivity, subtype selectivity, and/or species specificity in targeting various nAChRs.

### 2.12. Proline Residues Flanking Protein–Protein Interaction Sites

Previously, we showed that proline residues are commonly found in the flanking segments of protein–protein interaction sites [90]. Proline residue is unique with its side chain forming a cyclic five-membered ring structure with the main chain. Thus, when this amino acid makes a peptide bond, it lacks a free proton that could participate in hydrogen bonding in typical α-helices and β-sheets. Therefore, proline residue is a strong breaker of regular secondary structures like α-helices and β-sheets. Proline residues can promote bends and kinks in the polypeptide chain, facilitating the formation of turns. Hence, proline residues play two important structural roles in the flanking segments: (a) they protect the integrity and conformation of the interaction sites and (b) they help in the presentation of the protein–protein interaction sites by inducing kinks in the protein structures (for details, see [90]). Interestingly, two out of three segments (loops I and III) of the α-neurotoxin functional sites contain proline residues (Figure 1 and Figure 2). In both loops, the number of proline residues varies. At times, there are no proline residues. There are also several proline residues in the C-terminal tail of long-chain (Type II) neurotoxins. To understand the role of these proline residues in the conformation of the loops and the C-terminal tail, we predicted the 3D structures of several α-neurotoxins.

### 2.13. Predicted 3D Structures of α-Neurotoxins

**Figure 10:**
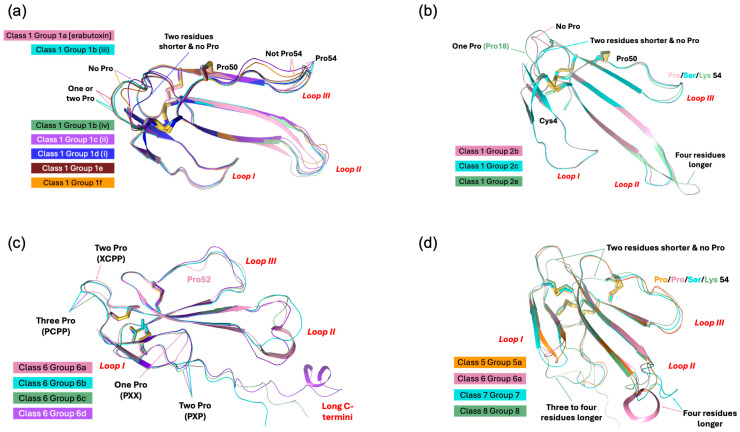
Structural alignment of Type I neurotoxins. (**a**) Superpositions of selected Class 1 Group 1 neurotoxins showing structural variations in loop III introduced by flanking Pro50 and Pro54. Changes in the number of residues and the presence of Pro also introduce variations in the connecting loop between loops I and II, although the functional effect of such changes may be minimal since this site is not directly involved in binding to nAChRs. Variations in the tip of loop II potentially contribute to nAChR subtype selectivity. Class 1 Group 1a is pink, Class 1 Group 1b (iii) is cyan, Glass 1 Group 1b (iv) is green, Class 1 Group 1c (ii) is purple, Class 1 Group 1d (i) is blue, Class 1 Group 1e is brown, Class 1 Group 1f is orange. (**b**) Superpositions of selected Class 1 Group 2 neurotoxins showing the effect of length and Pro in the connecting loop between loops I and II. Notably, Class 1 Group 2e has a longer loop II. Class 1 Group 2b is pink, Class 1 Group 2c is cyan, Class 1 Group 2e is green. (**c**) Superpositions of selected Class 1 Group 6 neurotoxins showed that the presence of flanking Pro in loop I affects the conformation of the loop. Class 6 Group 6a is pink, Class 6 Group 6b is cyan, Class 6 Group 6c is green, Class 6 Group 6d is purple. (**d**) Superpositions of selected Class 5 to 8 neurotoxins demonstrating diversity in the length of loops I and II. The crystal structure of a Class 6 Group 6a neurotoxin showed that the longer loop II has a similar conformation to that of a prototypical Type II neurotoxin (e.g., α-bungarotoxin/α-cobratoxin). Class 5 Group 5a is orange, Class 6 Group 6a is pink, Class 7 Group 7 is cyan, Class 8 Group 8 is green. Residues are numbered by homology numbering indicated in Figure 1, Figure 2, Figure 3, Figure 4, Figure 5, Figure 6 and Figure 7. PDB id for erabutoxin: 1QKD; Class 6 Group 6a: 8D9Y; structures of all other neurotoxins were predicted by AlphaFold3.

**Figure 11:**
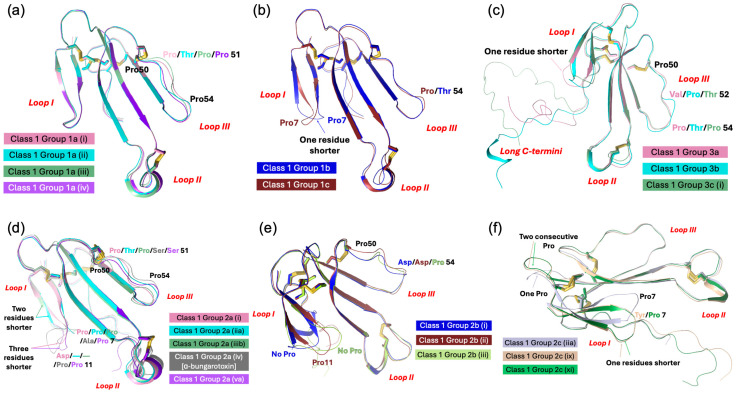
Structural alignment of Type II neurotoxins. (**a**) Superpositions of selected Class 1 Group 1 neurotoxins showing most variations occurred in loop III flanked by Pro50 and Pro54. Class 1 Group 1a (i) is pink, Class 1 Group 1a (ii) is cyan, Class 1 Group 1a (iii) is green, Class 1 Group 1a (iv) is purple. (**b**) Superpositions of selected Class 1 Group 1 neurotoxins showing a single residue deletion in loop I may drastically affect the conformation of the loop. Class 1 Group 1b is blue, Class 1 Group 1c is brown. (**c**) Superpositions of selected Class 1 Group 3 neurotoxins demonstrating the effect of variation in loop I size and flanking Pro in loop III. These neurotoxins also have long and variable C-termini. Class 1 Group 3a is pink, Class 1 Group 3b is cyan, Class 1 Group 3c (i) is green. (**d**) Superpositions of selected Class 1 Group 2 neurotoxins similarly showing the effect of variation in loop I size and flanking Pro. Variations in the tip of loop II potentially contribute to nAChR subtype selectivity. Class 1 Group 2a (i) is pink, Class 1 Group 2a (iia) is cyan, Class 1 Group 2a (iiib) is green, Class 1 Group 2a (iv) is grey, Class 1 Group 2a (va) is purple. (**e**) In some sequences, Pro in loop I further influences structural variation. Class 1 Group 2b (i) is blue, Class 1 Group 2b (ii) is brown, Class 1 Group 2b (iii) is lime. (**f**) Changes in the number of residues and the presence of Pro also introduce variations in the connecting loop between loops I and II, although the functional effect of such changes may be minimal since this site is not directly involved in binding to nAChRs. Class 1 Group 2c (iia) is pale blue, Class 1 Group 2c (ix) is wheat, Class 1 Group 2c (xi) is light green. Residues are numbered by homology numbering indicated in Figure 1, Figure 2, Figure 3, Figure 4, Figure 5, Figure 6 and Figure 7. PDB id for α-bungarotoxin: 1HC9; structures of all other neurotoxins were predicted by AlphaFold3.

**Figure 12:**
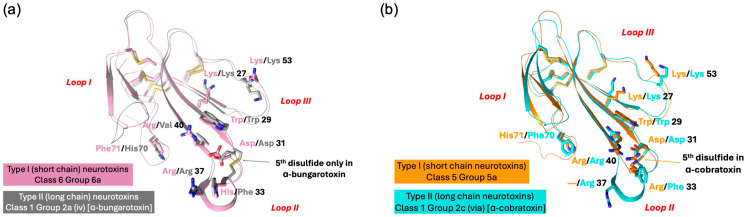
Conservation of functional residues in Type I and Type II neurotoxins. (**a**) Crystal structure of Type 1 Class 6 Group 6a (pink) superposes well on Type II (long chain, grey) neurotoxin, despite the missing ‘canonical’ fifth disulfide bridge in loop II. Many functional site residues in both these structures are topologically well conserved. (**b**) Most functional site residues in Type 1 Class 5 Group 5a (orange) neurotoxin superpose well on α-cobratoxin (cyan) residues, despite the absence of half-helix structure imposed by the fifth disulfide. Residues are numbered by homology numbering indicated in Figure 1, Figure 2, Figure 3, Figure 4, Figure 5, Figure 6 and Figure 7. PDB id for α-bungarotoxin: 1HC9; erabutoxin: 1QKD; α-cobratoxin: 7ULG; Type 1 Class 6 Group 6a: 8D9Y; structure of Type 1 Class 5 Group 5a was predicted by AlphaFold3.

We used AlphaFold3 to predict the structures of selected Type I (short chain) (Figure 10) and Type II (long chain) neurotoxins (Figure 11). Despite distinct structural changes among them, overall 3D structure remains the same. As described above, the functional site residues among these subgroups are expected to be topologically conserved (Figure 9). Such conservation helps various subtypes to retain their ability to bind to the target nAChRs. The subtle changes in these functional site residues probably afford the distinct subtype selectivity. One of the extremes can be observed in Classes 5, 6, and 7 in Type I (short chain) neurotoxins; they have 2-4-residue deletions in loop I and C-terminal extensions with His^71^ (Class 5) and Phe^71^ (Classes 6 and 7), similar to Type II (long chain) neurotoxins (Figure 1 and Figure 2). Most likely, the loss of receptor-binding residues in shorter loop I is compensated by C-terminal extension with His^71^/Phe^71^, which interact with nAChR [30]. Interestingly, Class 8 has both loop I functional site residues and the C-terminal extension (Figure 1). In Type I, Classes 5 and 8 neurotoxins, loop II is four residues shorter, similarly to short-chain neurotoxins. However, this gap is filled by small (Gly/Ser) amino acid residues that can offer conformational flexibility in Classes 6 and 7 neurotoxins (Figure 10d). The experimentally determined structure of Class 6, Group 6a neurotoxin (PDB ID: 8D9Y) superposes perfectly on Type II (long chain) neurotoxin, despite the missing ‘canonical’ fifth disulfide bridge in loop II (Figure 12a). The functional site residues in both these structures are topologically well conserved. The AlphaFold3 predicted structures of Groups 6b, 6c, and 6d also mimic Type II (long chain) neurotoxins (Figure 10c). The functional site residues in Class 5a neurotoxin superpose on α-cobratoxin residues, despite the absence of a half-helix structure imposed by the fifth disulfide (Figure 12b). This ‘canonical’ functional site is also conserved to an extent in non-conventional neurotoxins [13] (Figure 9). Thus, candoxin and other related neurotoxins use similar functional site residues to interact with nAChRs (see below).

### 2.14. Functional Site Residues in Other Neurotoxin Families

#### 2.14.1. Non-Conventional Toxins

Non-conventional toxins have the fifth disulfide bond in loop I, unlike Type II α-neurotoxins, which have the fifth disulfide bond in loop II. Because of the presence of this disulfide bond, the tip of loop I is turned away from the ‘body’ of the molecule [117,118]. We suspect that the functional site residues in this family are the same/similar to those in α-neurotoxins. This group contains candoxin, which potently inhibits muscle-type (IC_50_, 10 nM; highly reversible) and α7 (IC_50_, 50 nM; almost irreversible) nAChRs [13]. It has most of the functional site residues in loops II and III [Lys^47(55)^ is replaced by Ser^47(55)^]. The loop I of candoxin does not participate in its interaction with nAChRs. Hence, candoxin shows high affinity towards nAChRs. Other neurotoxins in this group include γ-bungarotoxin [112], and weak toxins [10,11]. γ-Bungarotoxin is a postsynaptic toxin with an intravenous LD_50_ of 0.15 mg/g in mice. The details of its effects on various nAChRs are not yet known. The tripeptide Arg-Gly-Asp sequence at loop II contributes to its ability to inhibit platelet aggregation [212] and integrin α5 [213]. When compared to candoxin, weak toxins have lost a few additional functional sites in loop II. This may explain the micromolar affinity of these toxins towards nAChRs. The basic residues Arg^31(33)^ and Arg^32(37)^ in loop II (highlighted in green, Figure 4) play a crucial role in binding to nAChRs and mAChRs [122,123]. Systematic site-directed mutation studies are needed to identify all amino acid residues that are involved in interaction with nAChRs.

#### 2.14.2. Ω-Neurotoxins

Ω-Neurotoxins belong to the short-chain 3FTx family. Their loops I and III are five and three amino acid residues shorter compared to Type I α-neurotoxins (Figure 5). Using site-directed Ala substitution of 12 amino acid residues (1, 9, and 2 residues in loops I, II and III, respectively), we identified the amino acid residues that contribute to the interaction of Oh9-1 (the first member of the Ω-neurotoxins family) with rat αβεδ and α3β2 nAChRs (for details, see [15]). The functional site residues involved in interaction are distinctly different (Figure 5). Further site-directed mutation studies are needed to elaborate the functional site residues involved in binding to nAChRs.

#### 2.14.3. Other Neurotoxins

The functional residues of other neurotoxins, such as Type III neurotoxins, Σ-neurotoxins, and monomeric and dimeric colubrid neurotoxins, are yet to be defined. There are minimal details regarding the pharmacological profiles that are required to assess the subtype, interface, and species specificity towards nAChRs of these neurotoxins. So far, only one member each from Σ-neurotoxins and monomeric and dimeric colubrid neurotoxins have been characterized for their functional properties and three-dimensional structure [16,114,115]. Structure–function relationships to understand the key amino acid residues involved in their interaction with nAChRs are needed in these neurotoxins.

### 2.15. Dimerization and Neofunctionalization

#### 2.15.1. Non-Covalent Dimers

Although most 3FTxs are monomers, κ-Bungarotoxins/κ-flavitoxins are dimeric toxins. Each monomer is structurally similar to long-chain neurotoxins. The crystal structure of κ-bungarotoxin indicates the presence of ten β-strands in the dimer, with an extended six-stranded antiparallel β-sheet that is shared between the two subunits [105]. The two subunits in a dimer exhibit major differences in the conformation of residues 27(29)-36(38) at the tip of loop II. Six main chain-main chain and three side chain hydrogen bonds, along with van der Waal’s interactions between residues Phe^48(52)^ and Leu^56(60)^, form the dimer interface [105]. Similarly to α-neurotoxins, the invariant Arg^34^ is located at the tip of the second loop for each subunit. Monomeric long-chain neurotoxins inhibit peripheral muscle-type and neuronal α7 and α9/α9α10 nAChRs. In contrast, κ-bungarotoxins antagonize only neuronal α3β2 and α4β2 nAChRs (for a review, see [6]). Thus, dimerization appears to affect the pharmacological profile of the toxin. DPP2d, a neurotoxin from *Dendroaspis polylepis polylepis* venom, crystallizes as a dimer [94] similar to κ-neurotoxins [105]. This non-covalent homodimer is formed via a network of van der Waals interactions, four main chain–main chain hydrogen bonds, two main chain–side chain hydrogen bonds, and two interlocked salt bridges [Glu^54(58)^–Lys^56(60)^]. However, in solution at 150 mM NaCl, it elutes as a monomer [94]. Salt at isosmotic concentrations appeared to break the salt bridges at the dimeric interface. DPP2d blocks human muscle-type (α1β1γδ) and neuronal α7 nAChRs but not α3-containing nAChRs. Thus, its pharmacological profile resembles typical Type II α-neurotoxins and not κ-neurotoxins.

Unlike the above Type II (long-chain) dimeric toxins, we characterized two new classes of neurotoxin dimers that are short-chain 3FTxs, haditoxin [97] and fulditoxin [16]. The quaternary structure of haditoxin homodimer is formed by antiparallel arrangement with the characteristic six-pleated sheets similar to κ-bungarotoxins [97]. The haditoxin dimer is stabilized by 14 (six main chain and eight side chain) hydrogen-bonding contacts and two hydrophobic clusters. Thus, haditoxin forms tighter dimers than κ-bungarotoxin (for more structural details, see [97]). Typical monomeric short-chain α-neurotoxins are selective and potent blockers of muscle nAChRs but ineffective at neuronal nAChRs [214], while long-chain α-neurotoxins block both muscle-type and α7 nAChRs [215]. Interestingly, haditoxin antagonizes human adult muscle-type and neuronal α7, α3β2, and α4β2 nAChRs. Thus, its nAChR subtype selectivity is distinctly different than monomeric short-chain (only muscle-type) and long-chain (muscle-type and α7) α-neurotoxins as well as dimeric κ-bungarotoxin (α3β2 and α4β2). Fulditoxin is a short-chain 3FTx [16]. It has four and three amino acid residues shorter loops I and III compared to typical short-chain neurotoxins. Fulditoxin does not form antiparallel dimers held together mostly by hydrogen bonding contacts between loop III residues. In contrast, fulditoxin forms a distinct dimer that is held together primarily by 29 hydrophobic interactions between amino acid residues from loop II, and only three side chain–side chain hydrogen bonds (for more structural details, see [16]). Functionally, fulditoxin is among the highly potent postsynaptic neurotoxins in CBCM preparations (IC_50_ values: fulditoxin, 27.8 nM; α-bungarotoxin, 11.4 nM; erabutoxin b, 31.7 nM) [16]. However, this neuromuscular block is highly reversible, almost similar to candoxin. Fulditoxin inhibits hα1β1εδ nAChRs (IC_50_, 2.56 μM; ~100 times lower than that for avian muscle nAChR), human α7 (IC_50_, 6.57 μM), hα4β2 (IC_50_, 1.8 μM), and hα3β2 (IC_50_, 12.6 μM) (for details, see [16]). Thus, fulditoxin dimer exhibits distinct subtype and species selectivity.

#### 2.15.2. Covalent Dimers

The covalent dimers are held together by disulfide bonds. They are isolated from *Naja kaouthia* venom [106] or colubrid snake venoms [115,216]. The *N. kaouthia* venom contains homodimers of α-cobratoxin (αCT-αCT) and heterodimers containing αCT and cytotoxins (Figure 2). The homodimerization of α-CT results in the blocking ability towards α3β2 nAChR with a loss of activity towards muscle-type and α7 nAChRs. Heterodimers of αCT with cytotoxins retain neurotoxic function on *Torpedo* and α7 nAChRs, but they lose their cytotoxicity [106]. Although these heterodimers have only one nAChR-binding loop II, they block α3β2 nAChR better than αCT-αCT [107]. Thus, dimerization results in distinct nAChR subtype selectivity. These cobra venom dimers are held together by two disulfide bonds by the loop I cysteine residues (Figure 2). This opens the loop I in both subunits with the formation of two antiparallel β-sheets between the two subunits.

Unlike heterodimers in the cobra venom, the ones in colubrid venoms are held together by a single disulfide bond between two new cysteine residues, one each in both subunits. Such cysteine residue is in the middle of loop II in the irditoxin A subunit, while it is in the middle of loop I in the irditoxin B subunit [115]. Irditoxin heterodimer exhibits ~1000-fold higher neurotoxicity at avian (e.g., CBCM) compared to its neurotoxicity at mammalian (e.g., rat hemidiaphragm) neuromuscular junction preparations. Sulditoxin, structurally similar to irditoxin, exhibits lizard-specific toxicity [216]. The pharmacological properties of individual subunits are not known.

## 3. Conclusions

Since α-bungarotoxin, the first postsynaptic neurotoxin from snake venom, was isolated and identified in 1963 [217], there have been many exciting discoveries of several new classes of neurotoxins that have been isolated and characterized. With the explosion of transcriptomic, proteomic, and genomic data, we have access to the sequences of a large number of toxins. Although α-neurotoxins are the largest and most well-studied family of neurotoxins, several new families of neurotoxins have been characterized in the last six decades. The neurotoxins described here share a common 3FTx protein fold, but they differ in their functional sites, subtype, and interface selectivity, and/or species specificity. They have evolved at different geological times and through unusual convergent evolution to target key receptors in neurotransmission, nAChRs. The non-exhaustive classification described here will help us recognize distinct structural and functional characteristics and provide impetus to study their pharmacological profiles and delineate their functional sites of various classes of neurotoxins. Such details will help us in designing subtype-, interface-, and species-selective ligands for nAChRs. Although some of these studies identify ligands that show low affinity, with better knowledge of structure–function relationships, it may be possible to enhance affinity and selectivity with a small number of mutations. For example, we used mutations of two amino acid residues in fasxiator (a factor XI inhibitor from *Bungarus fasciatus* venom) to improve its potency by ~1000 times [218,219]. Similarly, mutations of 1–2 residues have led to significant improvements in the activities of α-conotoxin AuIB [220] and mambalgin [221]. By engineering loops and/or loop tips of 3FTxs, chimeric toxins were designed with desired, enhanced activities. For instance, the grafting of the disulfide-bonded, cyclized loop II tip of a long-chain α-neurotoxin onto a short-chain toxin, Mourier et al. designed a chimera selective towards α7 nAChR [222]. Similarly, engineered chimeric toxins designed by substituting loops on toxin MT7 with those from toxin MT1 show impressive gain of function [223]. These results strongly support the possibility of achieving a new and original selectivity profile with high affinity for muscarinic receptor M1 and α_1A_-adrenoceptor. These observations indicate the opportunities to design new 3FTxs with novel pharmacological profiles, be it against nAChRs or other important therapeutic targets [224]. Furthermore, the exceptionally high affinity and specificity for diverse molecular targets, combined with their protease resistance, low immunogenicity, wealth of structural knowledge, and scalable production [225], make these small, disulfide-stabilized proteins an attractive protein scaffold for the design of exciting biomedical ligands with new functionality.

## 4. Methods

### 4.1. Messenger RNA and Protein Sequences

Messenger RNA and protein sequences of 3FTxs were downloaded from publicly available NCBI (National Center for Biotechnology Information) and UniProt databases. In most analyses, mature toxin sequences were used after removing the signal peptide (online SignalP 5.0 server) and propeptide regions. It is important to note that we selected toxins that are known as neurotoxins targeting nAChRs or that show sequence similarity with such toxins. The sequences were initially aligned based on BLAST output and subsequently subjected to further adjustment. Toxin classification was performed by manually grouping sequences with similar substitutions in functional residues and structurally important features, such as disulfide bonds, loop size, flanking prolines, C-terminal extension, and others.

### 4.2. Analysis of Functional Sites of Neurotoxins

The amino acid residues that are involved in the recognition and binding to nAChRs, forming the functional sites of various classes of neurotoxins, were identified by site-directed mutagenesis [15,16,27,28,29,30,31]. Based on the variation in these functional sites, neurotoxins were classified. Such classifications will provide impetus in studying the structure, function, and other details of unique members. We used three-dimensional structures of some of these 3FTxs that were determined by X-ray crystallography or NMR spectroscopic methods and deposited in the Protein Data Bank. We also used AlphaFold3 [33] to predict the three-dimensional structures of selected 3FTxs. These structures were visualized and analyzed using PyMOL software (The PyMOL Molecular Graphics System, Version 2.5.0 Schrödinger LLC, New York City, NY, USA). PyMOL was also used for preparing the protein structure figures.

## Figures and Tables

**Table 1:** Classification of snake venom three-finger neurotoxins.

Name	Class	Group	Total Number of Sequences	Subgroups	Main Grouping Criteria (Refer to Text for Details)
Type I, Short-chain neurotoxins	1	1a	11		Erabutoxin and closely related toxins
		1b	39	i–iv	Limited and conserved substitutions in functional residues
		1c	22	i–iii	Pro^12^ instead of Pro^11^
		1d	10	i–v	Pro^11^ and Pro^12^
		1e	8		Pro^7^ and Pro^11^
		1f	5		Only Pro^44(50)^
		1g	3		Gln10Thr substitution
		1h	4		Only 4 functional residues are retained
		1i	16		Only a few functional residues are conserved
	2	2a	1		Cys^4^ and Phe^32(36)^
		2b	17		Cys^4^ and His^32(36)^
		2c	11		Cys^4^ and Ser/Thr/His/Arg^48(54)^
		2d	2		Cys^4^, Lys27Met, Asp31Gly, Phe32(36)Ile, and Arg33(37)His
		2e	3		Cys^4^ and long Gly/Ser-rich loop II
	3	3a	2		Cys^4^ and Cys^16^
		3b	1		7 out of 10 functional residues conserved
	4		2		Asp^31^, Phe/His^32(36)^, and Arg^33(37)^ deleted
	5	5a	2		Shorter loop I, longer C-terminus, all functional residues except Phe/His^32(36)^ are conserved
		5b	3		
		5c	1		
	6	6a	2		Shorter loop I, longer loop II and C-terminus, no fifth disulfide in loop II unlike long-chain α-neurotoxins
		6b	2		
		6c	1		
		6d	1		
	7		3		Shorter loop I, longer loop II and C-terminus, no fifth disulfide in loop II unlike long-chain α-neurotoxins, His32(36)Thr/Ser
	8		1		Loop I and II are characteristic of short-chain α-neurotoxins but has longer C-terminus like long-chain α-neurotoxins
	9		4		Covalent homodimer, few conserved functional residues
Type II, Long-chain neurotoxins	1	1a	22	i–iv	α-bungarotoxin and closely related toxins
		1b	2		Asp27(31)Asn, Phe29(33)Trp, Lys49(53)Asn, His65(70)Tyr
		1c	3		Additional substitutions
	2	2a	47	i, iia–c, iiia–c, iv, va–d, via–d, vii–viii	Longer C-terminus (6–13 residues), conserved His^65(70)^, various other substitution among functional residues
		2b	6	i–iii	Longer C-terminus (6–13 residues), His/Phe^65(70)^ is non-conserved
		2c	43	i, iia–b, iiia–b, iva–b, v, via–c, viia–b, via–b, ix, xa–b, xi–xiii	Longer C-terminus (7–13 residues), conserved Phe^65(70)^, varying substitutions among functional residues
	3	3a	2		Long C-terminus (17–24 residues), variable C-terminus sequences
		3b	9		
		3c	7	i–ii	
	4		9		κ-neurotoxins, non-covalent homodimers
	5		4		Non-covalent homodimers, functionally similar to α-neurotoxins, C-terminal amidation
	6		6		α-cobratoxin, covalent homo/hetero-dimers
Type III neurotoxins	1	1a	35		Each loop is shorter, and none of the functional residues are conserved
		1b	2		
	2		1		Longer C-terminus
Non-conventional neurotoxins	1	1a	3		Candoxin and related toxins, 5th disulfide in loop I, many short-chain α-neurotoxin functional residues in loop II are conserved
		1b	4		
		1c	1		
	2		3		γ-bungarotoxin and related toxins, Arg^27(29)^, Trp^29(31)^, and Lys^47(55)^
	3	3a	5	i–iii	WTX and related toxins, Arg^31(33)^, Arg^32(37)^, and Arg^37(42)^ as important functional residues
		3b	9	i–iii	
		3c	10	i–v	
		3d	11	i–iv	
	4		4		Longer C-terminus, slightly altered location of the 5th disulfide
Ω-neurotoxins	1	1a	3		Short loop I and III, functional residues in short- and long-chain α-neurotoxins are not conserved
		1b	4		
		1c	2		
		1d	2		
	2		1		
Σ-neurotoxins	1	1a	8		Non-covalent dimer, five-residue extension at C-terminus, functional residues in α-neurotoxins and Ω-neurotoxins are not conserved
		1b	10		
		1c	1		
	2	2a	10		Shortest C-terminus among Σ-neurotoxins, conserved residues at dimer interface
		2b	1		
		2c	2		
	3		1		Two residues extension at C-terminus compared to class 2, conserved residues at dimer interface
Colubrid FTxs	1	1a	58	i–iv	N-terminal Gln modified to pyroglutamic acid, seven-residue N-terminal extension compared to typical 3FTxs, fifteen-residue propeptide with Xaa-Gln processing site
		1b	6		Tetrabasic propetide processing site
		1c	9		Dibasic propeptide processing site
		1d	1		Dibasic propeptide processing site, seventeen-residue N-terminal extension compared to typical 3FTxs
		1e	2		Monobasic propeptide processing site, six-residue N-terminal extension compared to typical 3FTxs
		1f	1		α-colubritoxin, unknown propeptide and processing site
	2		15		N-terminal Pro, five-residue N-terminal extension, eight-residue propeptide, RRKKK-P processing site
	3		1		N-terminal Pro, seven-residue N-terminal extension, eight-residue propeptide, KKK-P processing site
	4		9		N-terminal Pro, six-residue N-terminal extension, seven-residue propeptide, RR/KR-P processing site
	5		56		N-terminal Pro, seven-residue N-terminal extension, eight-residue propeptide, RK-P processing site
	6		2		N-terminal Pro, six-residue N-terminal extension, seven-residue propeptide, monobasic processing site
	7		2		one- or three-residue N-terminal extension, no propeptide
	8	8a	43	i–iv	Covalent heterodimeric 3FTxs (subunit A), additional Cys in loop II, varying proteolytic maturation characteristics
		8b	2		
		8c	2		
	9	9a	27		Covalent heterodimeric 3FTxs (subunit B), additional Cys in loop I, varying proteolytic maturation characteristics
		9b	3		
		9c	2		
		9d	2		
		9e	2		
	10	10a	2		Similar to Classes 8 and 9, additional Cys in both loop I and II
		10b	1		
	11		2		Two conserved Cys of typical nonconventional 3FTx missing, disulfide pairing pattern unknown

## Data Availability

No new data were created or analyzed in this study.
